# Genomewide Association Analyses of Lactation Persistency and Milk Production Traits in Holstein Cattle Based on Imputed Whole-Genome Sequence Data

**DOI:** 10.3390/genes12111830

**Published:** 2021-11-19

**Authors:** Victor B. Pedrosa, Flavio S. Schenkel, Shi-Yi Chen, Hinayah R. Oliveira, Theresa M. Casey, Melkaye G. Melka, Luiz F. Brito

**Affiliations:** 1 Department of Animal Sciences, Purdue University, West Lafayette, IN 47907, USA; vpedrosa@purdue.edu (V.B.P.); chen3505@purdue.edu (S.-Y.C.); holivier@uoguelph.ca (H.R.O.); theresa-casey@purdue.edu (T.M.C.); 2 Department of Animal Sciences, State University of Ponta Grossa, Ponta Grossa 84030-900, Brazil; 3 Centre for Genetic Improvement of Livestock, Department of Animal Biosciences, University of Guelph, Guelph, ON N1G2W1, Canada; schenkel@uoguelph.ca; 4 Farm Animal Genetic Resources Exploration and Innovation Key Laboratory of Sichuan Province, College of Animal Science & Technology, Sichuan Agricultural University, Chengdu 611130, China; 5 Department of Animal and Food Science, University of Wisconsin River Falls, River Falls, WI 54022, USA; melkaye.melka@uwrf.edu

**Keywords:** dairy cattle, extended lactation, genetic variants, genomewide association study, GWAS, QTL

## Abstract

Lactation persistency and milk production are among the most economically important traits in the dairy industry. In this study, we explored the association of over 6.1 million imputed whole-genome sequence variants with lactation persistency (LP), milk yield (MILK), fat yield (FAT), fat percentage (FAT%), protein yield (PROT), and protein percentage (PROT%) in North American Holstein cattle. We identified 49, 3991, 2607, 4459, 805, and 5519 SNPs significantly associated with LP, MILK, FAT, FAT%, PROT, and PROT%, respectively. Various known associations were confirmed while several novel candidate genes were also revealed, including *ARHGAP35*, *NPAS1*, *TMEM160*, *ZC3H4*, *SAE1*, *ZMIZ1*, *PPIF*, *LDB2*, *ABI3*, *SERPINB6*, and *SERPINB9* for LP; *NIM1K*, *ZNF131*, *GABRG1*, *GABRA2*, *DCHS1*, and *SPIDR* for MILK; *NR6A1*, *OLFML2A*, *EXT2*, POL*D1*, *GOT1*, and *ETV6* for FAT; *DPP6*, *LRRC26*, and the KCN gene family for FAT%; *CDC14A*, *RTCA*, *HSTN*, and *ODAM* for PROT; and *HERC3*, *HERC5*, *LALBA*, *CCL28*, and *NEURL1* for PROT%. Most of these genes are involved in relevant gene ontology (GO) terms such as fatty acid homeostasis, transporter regulator activity, response to progesterone and estradiol, response to steroid hormones, and lactation. The significant genomic regions found contribute to a better understanding of the molecular mechanisms related to LP and milk production in North American Holstein cattle.

## 1. Introduction

Milk production and composition are the most intensively selected traits in dairy cattle breeding programs around the world due to their direct economic impact to the industry and close link with nutritional properties [1,2]. Additionally, lactation persistency (LP), defined as the ability of a cow to maintain milk production at a high level after reaching the milk production peak, greatly impacts the economic return of the dairy sector [3]. Different indicators of LP have been proposed over time [4,5,6,7], with heritability estimates ranging from 0.14 to 0.24 [8,9]. Heritability estimates in Holstein cattle for milk (MILK), fat (FAT), and protein (PROT) yields usually range from 0.24 to 0.52, and from 0.36 to 0.68 for fat percentage (FAT%) and protein percentage (PROT%) [10,11,12,13].

Identifying genomic regions and candidate genes related to milk production traits is crucial to better understand the biological mechanisms underlying their phenotypic expression and to optimize genomic evaluation of milk-related traits [14]. In this context, genomewide association studies (GWAS) have been extensively performed in recent years to find associations between genomic polymorphisms and economically important traits in dairy cattle populations [15,16,17]. The large majority of these studies have been performed based on medium-density genotyping arrays. However, the identification of variants associated with target traits is highly dependent on the extent of linkage disequilibrium (LD) between the SNPs and the causal variants. Thus, an alternative to increase the ability to identify key genomic regions is the use of whole-genome sequence (WGS) data. More accurate quantitative trait loci (QTL), causative mutations, and consequently, candidate genes, are expected to be identified based on WGS [18]. Generating WGS data for a large number of animals is still expensive, but a technique known as genotype imputation can be employed to impute missing markers from animals genotyped with medium- or high-density genotyping arrays to WGS with accuracies greater than 90–95% [19]. The potential of imputed WGS (iWGS) data to discover genetic variants in GWAS has been shown in previous studies of dairy cattle [20,21] and other species [22,23,24].

New genomic regions associated with milk production traits in dairy cattle have been recently reported based on iWGS studies. For instance, Teissier et al. [25] identified 493 QTL for MILK, FAT, PROT, FAT%, and PROT% in Holstein, Montbéliarde, and Normande cattle breeds, demonstrating that a large number of genomic regions influence milk production traits. Furthermore, meta-analyses studies have reported pleiotropic effects of genes related to milk production traits and other dairy traits such as mammary system conformation and milking temperament [26,27]. In this context, genomewide fine mapping has been well explored for milk yield and milk solids [28,29,30]. However, to the best of our knowledge, there are no reports of LP-related genes identified using WGS or iWGS.

The LP reflects the cow’s ability to maintain milk production after the lactation peak, and may be an indicative of postcalving development of the mammary gland [31]. Improving LP can potentially increase cow health and welfare [9,32,33]. Despite the importance of LP to dairy cattle production, few studies have investigated its genomic background. Moreover, to our best knowledge, all previous studies used medium- to high-density SNP panels [34,35,36,37] instead of WGS or iWGS. Therefore, the main objectives of this study were to perform: (1) iWGS-based GWAS for LP, MILK, FAT, PROT, FAT%, and PROT% aiming to identify key genomic regions and candidate genes influencing these traits in North American Holstein cattle; and (2) functional genomic analysis to better understand the biological pathways associated with LP and milk production traits.

## 2. Materials and Methods

### 2.1. Animals and Phenotypes

All datasets (i.e., pedigree, phenotypes, and genotypes) were provided by the Canadian Dairy Network (CDN), a member of Lactanet (Guelph, ON, Canada). A range of 8264 (MILK) to 3447 (LP) animals with pseudo-phenotypes (de-regressed breeding values, dEBVs), for MILK, FAT, PROT, FAT%, PROT%, and LP traits were used in this study (Table 1). The phenotypes used in the genetic evaluations were obtained by the Dairy Herd Improvement (DHI, Canada) field staff in milk recording and posterior laboratory analyses. Official genetic evaluations for all evaluated traits and their associated reliabilities were provided by CDN (www.cdn.ca, accessed on 30 October 2021) based on their routine genetic evaluation models. The EBVs of LP were computed using the solutions from the Canadian test-day model, where an EBV for milk yield at day 60 and day 280 was calculated for each animal individually for each lactation (1, 2, and 3). Then, the EBVs were standardized to relative breeding values and combined into a single EBV (mean of 100 and standard deviation of 5), in which a higher EBV indicated a more persistent animal. The dEBVs were computed as in VanRaden et al. [38], and only dEBVs with a reliability greater than 0.28 (accuracies greater than 0.50) were kept for this study.

### 2.2. iWGS and Genomic Quality Control

Imputed WGS data from 9131 Holstein cows, with 29,548,077 SNPs were available for this study. Genotype imputation was performed first from medium-density SNP panels (MD, 9131 cows and 56,955 or 60,914 SNPs; Illumina, San Diego, CA, USA) to a high-density panel (HD, 311,725 SNPs; Illumina, San Diego, CA, USA); and secondly, from HD to WGS, resulting in the iWGS datasets. The HD reference population had 2397 animals (from the North American Holstein population), and there were 1147 animals with WGS data (from the 1000 Bull Genomes Project, which also included North American Holstein animals). Imputation was performed using the FImpute software [39]. Imputation accuracies greater than 95% have been achieved for Holstein and other North American dairy cattle breeds [19,40].

Before imputation, SNPs with a call rate lower than 0.95, extreme departure from Hardy–Weinberg equilibrium (*p* < 10^−8^), located in nonautosomal chromosomes or with unknown position, and excess of heterozygosity greater than 0.15 were removed from the dataset. Only SNPs present in both MD and HD files were retained in the MD dataset. After quality control (QC), 38,955 SNPs remained in the MD dataset to be used in the imputation analyses. A total of 297,114 SNPs (from the initial 311,725 SNPs) remained in the HD panel dataset after QC. Subsequently, imputation from HD to WGS was performed for all autosomal chromosomes.

After imputation to WGS, an additional QC was performed to remove the individuals and SNPs with a missing rate greater than 0.1, minor allele frequency (**MAF**) lower than 0.01, and extreme deviation from Hardy–Weinberg equilibrium (*p* < 10^−8^; e.g., [41,42,43]. Finally, a range of 5,108,861 to 6,101,357 SNPs remained for the GWAS analyses of LP and milk production traits. The PLINK software [44] was used to perform all the QC applied. All these analyses were done following Chen et al. [21].

### 2.3. Association Analyses, Statistical Models, and Significance Testing

Association analyses were performed using the GCTA package [45], fitting a mixed linear model (MLM), including a polygenic effect. Therefore, SNP effects were estimated using the following statistical model:

y=1μ+Xb+Zu+e,

where y is a vector of pseudo-phenotypes (dEBVs); 1 is a vector of ones; μ is the overall mean; b is the fixed effect of the SNP tested for association with each trait, **X** is a vector containing the genotype score for the tested SNP; u is a vector of polygenic effects with $u\;\sim \;N\left(0,\;G\sigma_{u}^{2}\right)$, where G is the genomic-based relationship matrix (GRM) and $\sigma_{u}^{2}$ is the additive genetic variance of polygenic effects; Z is the incidence matrix of u; and e is a vector of residual effects with $e\;\sim \;N\left(0,\;I\sigma_{e}^{2}\right)$, where I is an identity matrix and $\sigma_{e}^{2}$ is the residual variance.

To avoid double-fitting candidate SNPs [46], GRMs were alternatively constructed by randomly sampling 50,000 SNPs from all chromosomes, except the one in which the analyzed SNPs were located. After the GWAS analyses, all SNPs were ranked based on their *p*-values and clumped according to their LD pattern (*r*^2^  >  0.90), as suggested by Prive et al. [47]. The genomic inflation factor (λ) was calculated as the ratio of the median of the observed distribution of the $X^{2}$ statistic to the expected median $(\hat{\lambda }=median\left(X^{2}\right)/0.4549$), for which a 95% confidence interval (**CI**) of λ value was further derived.

To avoid the occurrence of excessive false-negative results by using the Bonferroni adjustment, as a large number of variants were tested [48], we alternatively calculated the threshold of significant testing as 0.05 divided by the number of independent chromosomal segments ($M_{e}$) at chromosome-wise levels [49]. $M_{e}$ is a function of the effective population size ($N_{e}$) and chromosome length (L, in centi-Morgans—**cM**), and was calculated as [50]:

$$M_{e}=\frac{2N_{e}L}{\log\left(N_{e}L\right)}$$

$N_{e}$ was considered to be equal to 58 [51] and one cM equivalent to 1 Mb [52]. The SNPs were considered as statistically significant if their $-\log_{10}\left(P\right)$ was higher than the chromosome-wide threshold.

### 2.4. Functional Genomic Analyses

The SNP coordinates were based on the ARS-UCD1.2 assembly of the cattle reference genome (GCA_002263795.2). The annotation information was obtained from the National Center for Biotechnology Information (NCBI; www.ncbi.nlm.nih.gov, accessed on 30 October 2021). The GALLO R package [53] was used to detect genes located within ±100 Kb of the significant SNPs and QTL regions previously cited in the Animal QTLdb [54]. The Variant Effect Predictor (VEP) tool from Ensembl [55] was utilized to identify novel variants associated with the main peaks observed in the Manhattan plots. Functional enrichment analyses of the candidate genes identified were performed using the DAVID platform [56]. Gene network joint analyses were performed using the STRING database [57].

## 3. Results

### 3.1. GWAS

**Figure 1:**
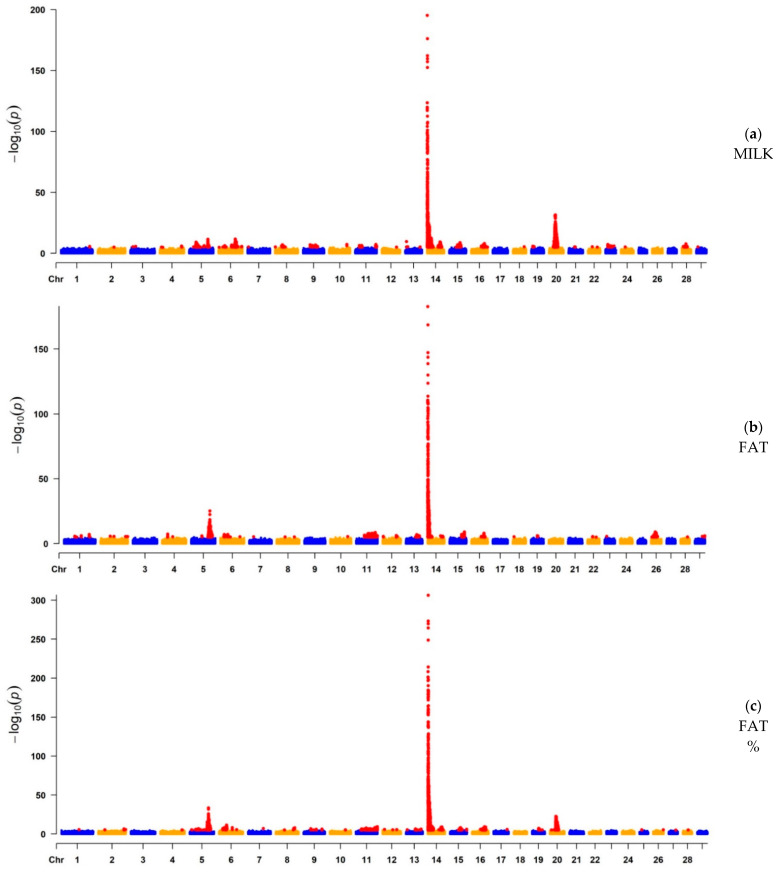

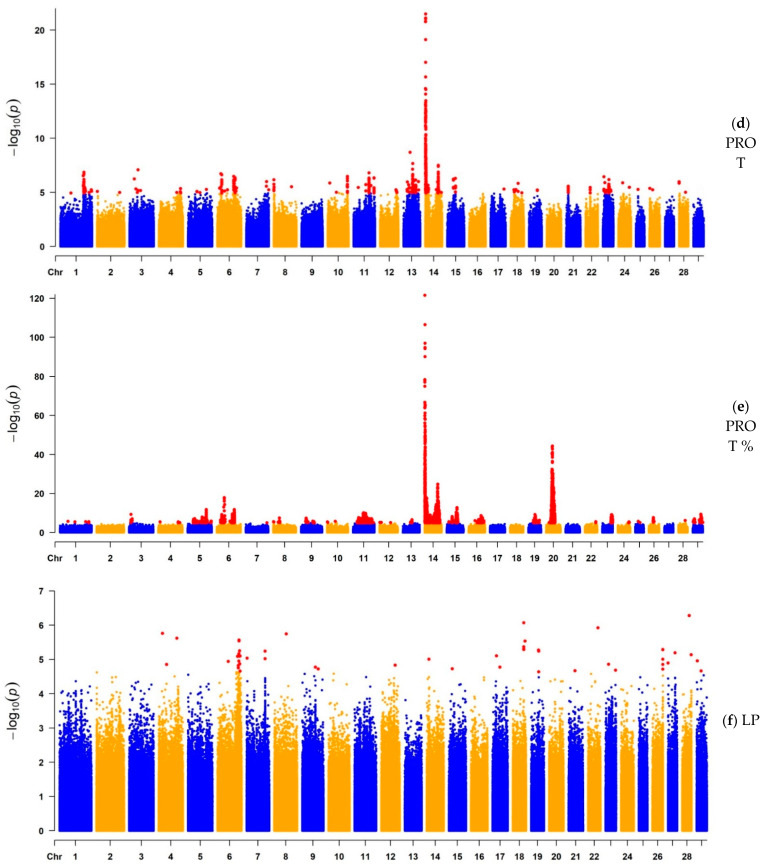
Manhattan plots of the GWAS results for milk yield (MILK), fat yield (FAT), fat percentage (FAT%), protein yield (PROT), protein percentage (PROT%), and lactation persistency (LP) based on imputed whole-genome sequence data. Statistically significant SNPs are represented by red dots.

After QC and LD-based clumping [47], the remaining number of informative SNP ranged from 1,673,052 (FAT) to 2,117,121 (LP). The Manhattan plots illustrate the chromosomal distribution of SNPs significantly associated with each trait (Figure 1). Additionally, Manhattan plots with a *y*-axis truncated at a lower level for the milk production traits are available as Figure S1 for a better visualization of peaks other than those found on BTA14. For the milk production traits, the significant peaks were higher and sharper, suggesting a more precise detection of narrower QTL regions distributed across the whole genome. A strong association was found in BTA14 (Table 2 and Table 3), with *ARHGAP39*, *bta-mir-2308*, *C14H8orf82*, *LRRC24*, *LRRC14*, *RECQL4*, *MFSD3*, *GPT*, *PPP1R16A*, *FOXH1*, *KIFC2*, *CYHR1*, *TONSL*, *VPS28*, *ENSBTAG00000053637,* *SLC39A4*, *CPSF1*, and *ADCK5* harboring the most significant SNPs for MILK, FAT, FAT%, and PROT%; and with *MAF1*, *SHARPIN*, *CYC1*, *GPAA1*, *EXOSC4*, *OPLAH*, *SPATC1*, *GRINA*, *PARP10*, *PLEC*, and *bta-mir-2309* being the most significant candidate genes for PROT. Milk production traits were highly associated with the diacylglycerol O-acyltransferase 1 (*DGAT1*) gene (Tables S1–S5), contributing to the highest peak found in BTA14. For LP, significant SNPs were spread across various chromosomes and with less-defined peaks. The most significant regions for LP were observed in BTA28 (*p*-value = 5.28 × 10^−7^) and BTA18 (*p*-value = 8.56 × 10^−7^), in which, for the BTA28 peak, the most significant SNPs were associated with *ZMIZ1* and *PPIF*; and for the BTA18 peak, with *ARHGAP35*, *NPAS1*, *TMEM160*, *ZC3H4*, and *SAE1* (Table 2). Moreover, the genes presented in Table 2 and Table 3 were previously reported in the Animal QTLdb to be associated with a large number of QTL regions. Most of these QTL are related to milk production traits, but there are some others linked to reproduction, health, production, and exterior (conformation and appearance).

#### 3.1.1. GWAS for Milk Yield, Fat Yield, and Fat Percentage

For MILK, 3991 SNPs, located on 25 chromosomes, were significantly associated with 1098 genes within ±100 Kb genomic regions. The genomic regions located on BTA5, BTA6, BTA13, BTA14, and BTA20 were the most significant ones (*p*-value < 10^−10^), where *MGST1* and *SLC15A5* (*BTA5*); *MOB1B*, *DCK*, and *SLC4A4* (*BTA6*); *ISM1* and *TASP1* (*BTA13*); and *NIM1K*, *ZNF131*, *ENSBTAG00000042376*, *ENSBTAG00000054352, ENSBTAG00000052195*, and *ENSBTAG00000051111* (*BTA20*), were the top candidate genes associated with those regions, in addition to those already mentioned above for the BTA14. For FAT, 2607 SNPs, located on 21 chromosomes, were significantly associated with 989 genes. Of those, the key candidate genes were *SLC15A5* (BTA5); *NR6A1*, *bta-mir-181a-2*, *bta-mir-181b-2*, *OLFML2A*, *WDR38*, *RPL35*, *ARPC5L*, and *GOLGA1* (BTA11); *ACCSL*, *ACCS*, and *EXT2* (BTA15); *CEP350* and *QSOX1* (BTA16); and *PKD2L1*, *SCD*, *bta-mir-12016*, *WNT8B*, *SEC31B*, *NDUFB8*, and *HIF1AN* (BTA26), where the genomic regions located on BTA5, BTA11, BTA14, BTA15, BTA16, and BTA26 were the most significant ones (*p*-value < 10^−9^). Furthermore, for FAT%, 4459 SNPs, located on 22 chromosomes, were significantly associated with 2016 genes within ±100 Kb genomic regions. For this trait, the chromosomes with the most significant regions (*p*-value < 10^−10^) were BTA5, BTA6, BTA11, BTA14, BTA16, and BTA20. The top candidate genes located in those regions were *SLC15A5* (BTA5); *HERC3*, *PIGY*, and *HERC5* (BTA6); *GADD45G* (BTA11); *FAM163A*, *TOR1AIP2*, *TOR1AIP1* (BTA16); and *ENSBTAG00000054476* and *MRPS30* (BTA20). The top candidate genes located on BTA14 for MILK, FAT, and FAT% were *ARHGAP39*, *bta-mir-2308*, *C14H8orf82*, *LRRC24*, *LRRC14*, *RECQL4*, *MFSD3*, *GPT*, *PPP1R16A*, *FOXH1*, *KIFC2*, *CYHR1*, *TONSL*, *VPS28*, *ENSBTAG00000053637*, *SLC39A4*, *CPSF1*, and *ADCK5*. VEP analysis confirmed *FOXH1* as a gene containing variants strongly related to MILK, FAT, and FAT%. Furthermore, VEP analysis indicated *OLFML2A* (BTA11) as a possible novel candidate variant associated with FAT (Table S6).

#### 3.1.2. GWAS for Protein Yield, Protein Percentage, and Lactation Persistency

A total of 805 SNPs, located on 24 chromosomes, were significantly associated with 898 genes for PROT. Of those, BTA3, BTA13, and BTA14 contained the most significant regions (*p*-value < 10^−8^), where *CDC14A*, *ENSBTAG00000054319*, *ENSBTAG00000015759*, and *RTCA* (BTA3); *ARHGAP12* (BTA13); and *MAF1*, *ENSBTAG00000051469*, *SHARPIN*, *CYC1*, *GPAA1*, *EXOSC4*, *OPLAH*, *ENSBTAG00000015040*, *SPATC1*, *GRINA*, *PARP10*, *PLEC*, and *bta-mir-2309* (BTA14) were the candidate genes related to the most significant SNPs identified for PROT. Moreover, for PROT%, 5519 SNPs were located on 24 chromosomes, significantly related to 2739 genes within ±100 Kb genomic regions. The top significant genes for PROT% (*p*-value < 10^−12^) were *SLC15A5* (BTA5); *HERC3*, *PIGY*, and *HERC5* (BTA6); *ARHGAP39*, *bta-mir-2308*, *C14H8orf82*, *LRRC24*, *LRRC14*, *RECQL4*, *MFSD3*, *GPT*, *PPP1R16A*, *FOXH1*, *KIFC2*, *CYHR1*, *TONSL*, *VPS28*, *ENSBTAG00000053637*, *SLC39A4*, *CPSF1*, and *ADCK5* (BTA14); *STIM1*, *RHOG*, *PGAP2*, *NUP98* (BTA15); and *PAIP1*, *ENSBTAG00000049623*, *C20H5orf34*, *TMEM267*, *CCL28*, *HMGCS1*, *ENSBTAG00000048672*, and *NIM1K* (BTA20). Finally, for LP, 49 SNPs located on 18 chromosomes were significantly associated with 85 genes (Table S7). The most significant genes (*p*-value < 10^−7^) were located on BTA18 and BTA28, represented by *ARHGAP35*, *NPAS1*, *TMEM160*, *ZC3H4*, *SAE1* (BTA18); and *ZMIZ1* and *PPIF* (BTA28) (Table 3). The VEP analysis indicated the genes *GRINA* and *PARP10* for PROT, *CCL28* for PROT%, and the genes *ZC3H4* and *ZMIZ1* for LP (Table S6), all of which were located in the main observed GWAS peaks.

### 3.2. Commonly Identified Genes for Two or More Traits

**Figure 2:**
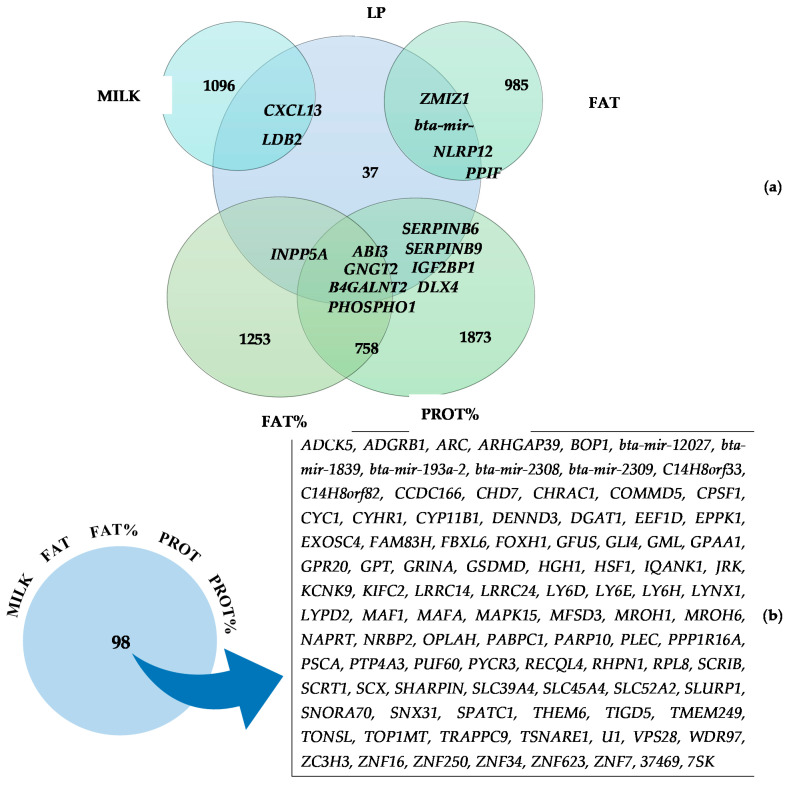
Number of genes and list of common genes related to lactation persistency (**a**) and associated between production traits (**b**), considering milk yield (MILK), fat yield (FAT), fat percentage (FAT%), protein yield (PROT), protein percentage (PROT%) and lactation persistency (LP).

Similar genomic regions were detected to be associated with different LP and milk production traits (Figure 2), indicating potential pleiotropic effects. LP presented common candidate genes with MILK, FAT, FAT%, and PROT%, where 15 candidate genes were concurrently associated with LP and the mentioned production traits: *CXCL13* and *LDB2* (MILK); *ZMIZ1*, *bta-mir-371*, *NLRP12* and *PPIF* (FAT); *INPP5A* (FAT%); and *SERPINB6*, *SERPINB9*, *IGF2BP1*, and *DLX4* (PROT%). Additionally, LP, FAT%, and PROT% showed common candidate genes between the three traits simultaneously: *ABI3*, *GNGT2*, *B4GALNT2*, and *PHOSPHO1*, demonstrating their importance not only in the genetic background of milk solids production, but also in the duration of the lactation peak.

As a result of the high genetic correlation existing between the milk production traits, 98 genes were commonly associated with the five milk production traits (MILK, FAT, FAT%, PROT, and PROT%), as demonstrated in Figure 2. All those common genes were located on BTA14, reinforcing the impact of this genomic region on milk production traits. The candidate genes located closer to genomic region linked to the top SNP found (BTA14: 465,742 bp) among MILK, FAT, FAT%, and PROT% were *PPP1R16A*, *FOXH1*, *KIFC2*, and *CYHR1*. In addition, the closer common genes related to the top SNP found for PROT (BTA14, BP= 827,938) were *GRINA*, *PARP10*, and *PLEC*.

**Figure 3:**
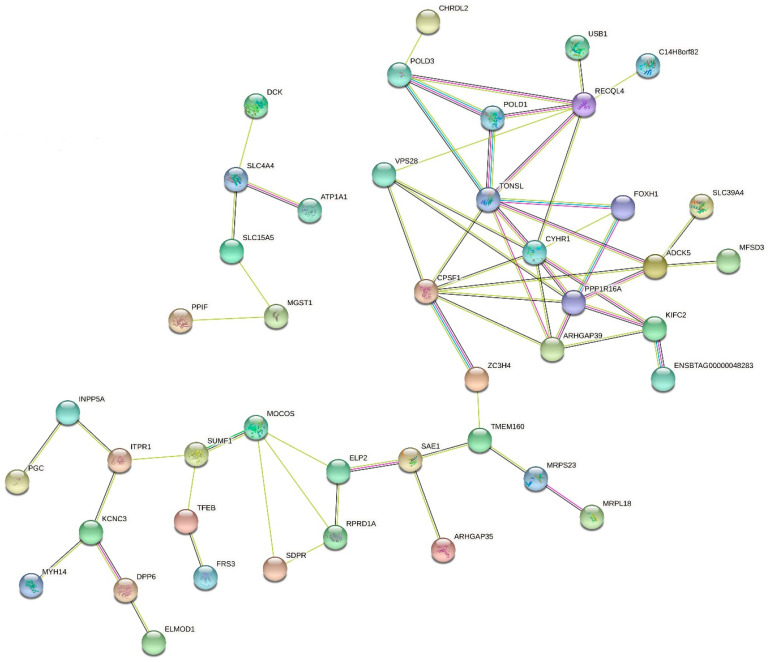
Gene interaction network for the central genes associated with lactation persistency, milk yield, fat yield, fat percentage, protein yield, and protein percentage in North American Holstein cattle.

The gene interaction network analysis revealed a strong connection between LP and milk production traits (Figure 3). Genes such as *ARHGAP35*, *TMEM160*, and *SAE1* (BTA18), which were highly associated with LP, demonstrated to be linked with *ARHGAP39*, *PPP1R16A*, *FOXH1*, and *CYHR1* (BTA14), which were significantly associated with milk production. Three big clusters were formed rounding *ARHGAP39*, *TONSL*, *ADCK5*, reinforcing that the molecular interactions among these three genes seem to be related to the control of the gene expression and protein regulation of milk traits.

### 3.3. Functional Analyses of Candidate Genes

Gene ontology (GO) enrichment analyses were performed to better understand the functional role of the candidate genes identified. GO terms for 14 biological processes and 12 molecular functions were significantly enriched, with 44 genes for MILK, 86 genes for FAT, and 33 for FAT% (Table 4). Furthermore, GO terms for 26 biological processes and nine molecular functions were significantly related to 41 genes for PROT, 109 genes for PROT%, and 6 for LP (Table 5). Five GO terms were found to be related to two or more traits. The GO:1903494 term associated with the response to dehydroepiandrosterone, GO:1903496 linked to the response of 11-deoxycorticosterone, and GO:0032355 associated with the response to estradiol were commonly identified for MILK, PROT, and PROT%. In addition, GO:0015125, related to bile acid transmembrane transporter activity, was identified for FAT and FAT%; and GO:0005149, which is related to the interleukin-1 receptor binding, was associated with FAT% and PROT%.

The following 26 genes were identified as influencing the phenotypic expression of two or more traits: *CSN1S1*, *CSN1S2*, *CSN2*, *CSN3*, *CHD7*, *KCNMB4*, *SLCO1A2*, *SLCO1B3*, *SLCO1C1*, *SLCO2B1*, *HCK*, *PTK2*, *SCX*, *DDX1*, *TNFRSF1A*, *LTBR*, *IL1A*, *IL1B*, *IL1F10*, *IL1RN*, *IL36RN*, *IL36A*, *IL36B*, *IL36G*, *IL37*, and *SERPINB9*. Furthermore, some genes were repeated in certain GO terms for the same trait, including the *GABR*, *CSN*, and *GST* gene families, *CLIC5*, and *MGST1* (*MILK*); *EYA3*, *DHCR7*, *PPDPF*, and *GADD* gene family (FAT); the *LYSB* gene and the *BPI*, *LYZ*, and *CSN* gene families (PROT), and *C1QBP*, *ID2*, *JMJD6*, *LALBA*, *MAGOHB*, *PABPC1*, *PRLR*, P*RPF4B*, *PUF60*, *SRSF2*, *SRSF7*, *STAT5B*, *WNT11*, and *ZPR1* genes, as well as *CSN*, *SLC*, and *RBM* gene families (PROT%).

## 4. Discussion

The majority of previous GWAS carried out to search for genomic regions associated with economically important traits in dairy cattle have been conducted using MD or HD SNP panel data. However, iWGS can increase the statistical power for detecting QTLs and causative variants for complex traits [58]. Based on iWGS-based GWAS, we identified novel genomic regions of interest, revealing novel candidate genes (*ARHGAP35*, *NPAS1*, *TMEM160*, *ZC3H4*, *SAE1*, *ZMIZ1*, *PPIF*, *LDB2*, *ABI3*, *SERPINB6*, and *SERPINB9* for LP; *NIM1K*, *ZNF131*, *GABRG1*, *GABRA2*, *DCHS1*, and *SPIDR* for MILK; *NR6A1*, *EXT2*, POL*D1*, *GOT1*, and *ETV6* for FAT; *DPP6*, *LRRC26*, and the KCN gene family for FAT%; *CDC14A*, *RTCA*, *HSTN*, and *ODAM* for PROT; and *HERC3*, *HERC5*, *LALBA*, and *NEURL1* for PROT%), and confirmed previously reported associations. The fairly high number of novel candidate genes for LP and milk production traits supports the use of a denser mapping of the genome for GWAS purposes, as well as the polygenic nature of these traits. Liu et al. [59] evaluating 1220 Holstein cows with a SNP panel containing 124,743 markers identified 10 highly significant SNPs associated with FAT and PROT. However, those authors did not detect any significant SNP related to milk yield, even with a moderate number of markers involved in the GWAS, reinforcing the argument that the use of WGS can substantially contribute to this type of analysis. For instance, numerous QTLs were found for MILK (146), FAT (152), and PROT (166) in five French and Danish dairy breeds when using WGS data [18]. Using iWGS-based GWAS, we identified multiply genomic regions affecting LP, a complex trait with few known QTLs. Therefore, our findings greatly contribute to elucidating the genetic background of LP in Holstein cattle.

### 4.1. Candidate Genes for Lactation Persistency

Among the traits evaluated in this current study, LP is the less explored one, and to our best knowledge, this is the first iWGS-based GWAS for LP, which presents a good opportunity to explore novel genomic regions influencing its phenotypic expression. Despite the fact that the peaks were not well defined due to the highly polygenic nature of LP [7], BTA18 and BTA28 were the ones with the most significant regions. The genes highly associated to LP on BTA18 were *ARHGAP35*, *NPAS1*, *TMEM160*, *ZC3H4*, and *SAE1* and on BTA28 were *ZMIZ1* and *PPIF*. None of these genes were previously linked to LP. Interestingly, most of these genes mentioned above associated with LP were previously linked to fertility traits in dairy cattle [60,61,62], indicating that fertility and LP are genetically correlated in dairy cattle populations [63]. Other studies have also demonstrated the genetic relationship between reproductive traits and LP in dairy cattle. For instance, Muir et al. [64] showed that heifers with difficult first calving tended to have more persistent first lactations and lower peak yields, indicating an antagonistic relationship between calving ease and LP. More recently, Yamazaki et al. [9] reported that differences in LP are related to a cow’s ability to conceive after the second calving. Therefore, the best bulls for improving female fertility after the second calving may differ with the production system and herd milk production, given that a strong genetic correlation was verified between LP and fertility traits in Japanese Holstein cattle [9]. Another important finding was the gene network connection among *ARHGAP35*, *TMEM160*, and *SAE1* (BTA18) and *ARHGAP39*, *PPP1R16A*, *FOXH1*, and *CYHR1* (BTA14), as demonstrated in Figure 3. No other study has reported a close network interaction between some of the main genes responsible for LP and milk production traits, indicating potential pleiotropic effects (Figure 3 and Supplemental Figure S2). Further studies should investigate the connection between LP and milk production traits at the molecular level. Evidence of interaction between LP and milk production traits were previously reported by Jakobsen et al. and Yamazaki et al. [65,66], where moderate to high genetic correlations were observed between these two trait groups.

Three GO terms were significantly enriched for LP, GO:0030334 (regulation of cell migration), GO:19003955 (positive regulation of protein targeting to mitochondrion), and GO:0004867 (serine-type endopeptidase inhibitor activity). The genes linked to the GO terms *ABI3* and *LDB2* (GO:0030334), *SAE1* (GO:1903955), and *SERPINB6* and *SERPINB9* (GO:0004867) could also be considered as novel candidate genes and its molecular role related to LP should be deeper investigated. The *ABI3*—ABI gene family member 3 was significantly associated in our study not only with LP but also FAT% and PROT% (Figure 2), confirming its influence in the phenotypic expression of bovine milk-related traits. The *ABI3* gene was previously related to milk pregnancy-associated glycoproteins in Holstein cattle but its molecular role in dairy cattle has been little explored [67]. *SAE1*, which also appear as one of the most significant genes for LP, was previously associated with lactation evolution in mice, indicating that this gene plays an important role in the expression of lactation in other mammalian species [68]. *SERPINB6* and *SERPINB9* are genes belonging to the serpins superfamily of protease inhibitors, which uses a conformational change to inhibit target enzymes. They are central genes controlling many proteolytic cascades, including important mammalian coagulation pathways [69]. Both genes were formerly associated with milk production traits in buffaloes [70] and somatic cell count in Jersey cows and clinical ketosis lactation in Holstein cattle [71,72], but this is the first time that *SERPINB6* and *SERPINB9* have been associated with LP. Due to its relationship with other dairy cattle traits, its molecular role in the expression of LP should be further investigated.

### 4.2. Candidate Genes for Milk Yield

Several powerful associations detected here support previously reported genomic regions for milk production traits. For instance, the region containing highly significant SNPs on BTA14 for MILK, including *ARHGAP39*, *PPP1R16A*, *FOXH1*, *KIFC2*, *CYHR1*, and *TONSL* was reported in other studies [15,37,73,74]. Atashi et al. [74] reported this region on BTA14 to be associated with 305-day milk yield and peak yield in dairy cows. Other relevant genomic regions were found on BTA20, BTA5, and BTA6, also containing genes previously identified as potentially influencing milk production. *MGST1*, *SLC15A5* (BTA5); *MOB1B*, *DCK*, *SLC4A4* (BTA6) and *NIM1K*, *ZNF131* (BT20) can be highlighted for its strong significance with MILK (Table 2). Raven at al. [75] reported the link between *MGST1* and milk production in multibreed cattle. Additionally, it is noteworthy that the *SLC4A4* gene had an important function in the production of MILK in a study with Holstein cattle in the USA [73]. *SLC4A4* is a solute transporter, belonging to one of the major transporter superfamilies mostly involved in the active transport of glucose. Glucose uptake by mammary epithelial cells is a crucial stage in milk synthesis, and therefore, directly influences MILK [76]. Furthermore, *NIM1K* and *ZNF131* have been reported to prolong lactation period culminating in higher milk production levels in Canadian dairy cattle [36]. Banos et al. [77] reported that *ZNF131* was expressed in the milk transcriptome and the mammary gland of dairy sheep, highlighting the impact of this gene in the process of molecular transcription of regions related to sheep milk production. The importance of zinc finger protein 131 (*ZNF131*) on the transcription of molecular codes responsible for the milk production seems clear. Lastly, this is the first time that *NIM1K* and *ZNF131* have been directly associated with milk yield in dairy cattle.

Four other novel candidate genes for MILK presented significant GO terms (FDR ≤ 1%) in the enrichment analyses. GO:0004890 and GO:0005230: *GABRG1* (BTA14) and *GABRA2* (BTA6); GO:0003273: *DCHS1* (BTA15) and GO:0071479: *SPIDR* (BTA14), shown in Table 4, can be highlighted as genes that might play fundamental roles in metabolic functions involving MILK. Interestingly, the four genes were previously linked to FAT [62,78,79] or other milk components [80], confirming its close relationship with milk production traits. For instance, γ-aminobutyric acid type A genes (*GABRG1* and *GABRA2*), which contributes to γ-aminobutyric acid (GABA) chloride ion channel activity and participates in GABA-A receptor activity, were previously associated with milk production traits in Holstein and Xinjiang Brown cattle [81,82].

### 4.3. Candidate Genes for Fat Yield

As observed for MILK, the GWAS for FAT revealed several genomic regions with highly significant SNPs associated with 989 genes spread across 21 chromosomes. Besides the genes located in BTA14 (ARHGAP39, *PPP1R16A*, *FOXH1*, *KIFC2*, *CYHR1*, and *TONSL*) identified for MILK, a highly significant genomic region was identified for FAT on BTA5, which harbors the *SLC15A5* gene. *SLC15A5* was previously associated with FAT, presented in a large region of 88.26–93.69 Mb on BTA5, that seems to have a cluster of additive effects linked with *MGST1*, *PLEKHA5*, and *ABCC9* [62]. The same genes (*MGST1*, *PLEKHA5*, and *ABCC9*) were also significantly associated with FAT in our study (Supplemental Table S1), suggesting that this genomic region plays an important role in the expression of FAT in Holstein cattle. Other significant peaks for FAT were observed on BTA11, BTA15, BTA16, and BTA26, revealing novel candidate genes. On BTA11, the most significant region contains the *NR6A1* gene, which was also included in the significant GO:0000978, demonstrating its potential for being a novel candidate gene associated with FAT. *NR6A1* has high homology among different species [83], and acts in the expression of traits linked to metabolism, reproduction, and production, as demonstrated in a study with swine where this gene was related with fat deposition [84]. Another relevant gene located on BTA11 that might be interesting to investigate further is *OLFML2A.* Besides been highly associated with FAT, *OLFML2A* was also found in VEP analysis, confirmg its potential as a novel candidate gene associated with FAT. *OLFML2A* (Olfactomedin Like 2A) is a protein coding gene related to protein homodimerization activity and extracellular matrix binding. To our best knowledge, this gene has never been associated with any milk trait in the literature before and its influence on milk related traits warrants a deeper investigated. Interestingly, the *OLFML2A* gene was found differently expressed in a study of fat depot-specific gene signatures in mice, contributing to the distinct patterns of extracellular matrix remodeling and adipose function in different fat depots [85]. Additionally, on BTA15, the most significant SNP found was associated with *EXT2*, which was also related with a significant GO term responsible for cell differentiation (GO:0030154). *EXT2* was previously cited as a suggestive gene associated with milk iron content in Jersey cows [86]. However, this is the first time that this gene has been directly associated with FAT in Holstein cattle.

Among the most significant GO terms, GO:0055089 is involved in fatty acid homeostasis in which important genes such as *DGAT1* are included. The link between the *DGAT* gene with FAT and other milk production traits is widely known [28,87]. However, out of the five genes related to GO:0055089, *POLD1* and *GOT1* have not been previously associated with FAT. *POLD1*, which is located on BTA18, was previously associated with PROT in Nordic Holstein cattle, but its function in the expression of milk production traits is not yet well established [88]. *GOT1*, located on BTA26, was previously associated with milk fatty acids acting in the transformation of citrate by ATP-citrate lyase in the cytosol, which is required for fatty acid synthesis [89]. Another novel candidate gene for FAT is *ETV6*, which was identified in two GO terms (GO:0030154 and GO:0000978), representing cell differentiation and RNA polymerase II core promoter proximal region sequence-specific DNA binding, respectively. *ETV6* was also previously associated with FAT% in Brown Swiss cattle [90] and MILK in Holstein cattle [78], but this is the first time that *ETV6* was linked to FAT.

### 4.4. Candidate Genes for Fat Percentage

For FAT%, 4459 SNPs, distributed across 22 chromosomes, associated with 2016 annotated genes were found. Among the highly significant regions, it can be highlighted the *SLC15A5* (BTA5) and *MRPS30* (BTA20) genes, besides those already mentioned from the BTA14 also found in MILK and FAT. *SLC15A5*, a solute carrier family member, was also recently associated with FAT% in Holstein cattle [62,91], confirming its importance to milk quality. Furthermore, other genes from the solute carrier family demonstrated to be relevant for FAT% since *SLCO1A2*, *SLCO1B3*, *SLCO1C1* and *SLCO2B1* were associated in the GO:0015125, one of the significant GO terms enriched for this trait (Table 4). *MRPS30* was first associated with FAT% in Jiang et al. [62] and previously linked to MILK in studies reported by Fang et al. and Cai et al. [92,93].

The most significant GO terms for FAT% are GO:0005149 (interleukin-1 receptor binding) and GO:0015459 (potassium channel regulator activity). The GO:0005149 presented the highest significance (8.5 × 10^−8^) and showed a big group of IL family genes associated to FAT% (Table 4). There are few reports in the literature integrating the interleukin-1 receptor with dairy cattle—only research that associated genes from this gene family with mastitis or fertility indicators [94,95]. However, there is proven evidence in other species, including humans, of the relationship between the IL gene family and the structuring of fat in different tissues, which may demonstrate the importance of some of these genes in the context of the structuring of milk fat [96,97]. Another important GO term found was the GO:0015459, which is related to the potassium channel activity. The main genes involved in this GO are *DPP6* (BTA4), *LRRC26* (BTA11) besides others from the KCN gene family. Genes from the KCN family were previously associated with milk traits [98], but its molecular involvement with FAT% needs to be further explored. A possible mechanism that might be worth investigating is that, in milk, potassium is correlated with lactose, and therefore with milk yield, via osmotic regulation [99]. Therefore, changes in percentage traits could be driven by potassium-induced changes in milk volume.

### 4.5. Candidate Genes for Protein Yield

For PROT, *CDC14A* and *RTCA* (BTA3); and *MAF1*, *SHARPIN*, *CYC1*, *EXOSC4*, *PARP10*, *OPLAH*, *GRINA*, *PLEC* (BTA14) are key candidate genes for milk protein expression. On BTA3, this is the first time that *CDC14A* and *RTCA* are associated with PROT, but interestingly both genes were already detected in signatures of selection of other milk production traits in Valdostana cattle populations [100]. Furthermore, *RTCA* was previously associated with milk production traits in buffalo [101], but its connection with milk traits has not been described yet. The *MAF1* gene located on BTA14 has been associated with milk protein synthetic capacity and for this reason, it has been pointed out as a key candidate genes for PROT [34,62,102]. The other genes identified on BTA14 (*SHARPIN*, *CYC1*, *EXOSC4*, *PARP10*, *OPLAH*, *GRINA*, and *PLEC*) were recently strongly associated with milk production traits, including PROT [37,93,102]. According to Jena et al. [103], *SHARPIN* influences mammary gland development and controls extracellular matrix organization of stroma during branching morphogenesis, which induces alveologenesis during pregnancy and lactation. Moreover, Lin et al. [104] found strong association of *SHARPIN*, *CYC1*, *EXOSC4*, and *PARP10* with milk serum albumin, which is one of the main protein contents of cattle milk. These facts reinforce the hypothesis that the large genomic region where these genes are located (0.5 Mb upstream and downstream from *SHARPIN*) is important for milk protein expression. It is important to highlight that both *GRINA* and *PARP10* were found in our VEP analysis, confirming that those two genes are highly associated with PROT and should be considered as novel relevant variants for this trait. The Glutamate Receptor Ionotropic NMDA-Associated Protein 1 (*GRINA*) belongs to the Lifeguard family and is involved in calcium homeostasis [105]. This gene was previously reported to play an important role in the lipid, major proteins, and cholesterol homeostasis in milk content of dairy cows, suggesting that *GRINA* might contribute to the regulation of solids present in dairy milk [12,102]. *PARP10*, which is a member of the poly (ADP-ribose) polymerases family, is related to several essential biological functions, such as immunity, metabolism, apoptosis, and DNA damage repair [106]. From a physiological perspective, the concentration of albumin in milk is influenced by pathological and genetic factors, which could connect the action of *PARP10* on the regulation of albumin in dairy cattle milk content [104].

The GO enrichment analyses for PROT (Table 5) also revealed the casein gene cluster containing the *CSN1S1*, *CSN1S2*, *CSN2*, and *CSN3* genes, which encode αs1, αs2, β, and κ casein, respectively. These genes have a strong influence on casein synthesis in cattle milk, and polymorphisms in this region have significant impact on milk protein composition [107]. In this context, *HSTN* and *ODAM*, which are located at the same region of the casein gene cluster, can be considered as novel candidate genes for PROT due to their close binding on BTA6 with *CSN1S1*, *CSN1S2*, *CSN2*, and *CSN3*, where all these genes are in linkage disequilibrium.

### 4.6. Candidate Genes for Protein Percentage

With several genomic regions adjacent to those mentioned in the other milk production traits, PROT% was the trait with the largest number of significant markers, i.e., 5519 SNPs, spread across 24 chromosomes and 2739 annotated genes. The most significant regions were found on BTA5, BTA6, BTA14, BTA15, and BTA20, with special emphasis on BTA6, BTA20, and BTA14. The most significant genes located on BTA6 are *HERC3*, *HERC5*, and *PIGY*. *HERC3* and *HERC5* can be considered as novel candidate genes for PROT% as they were previously associated with PROT and FAT%, respectively [29,30]. Genes from the HERC family of ubiquitin ligases associate with prolactin to regulate important milk proteins such as β-casein [108], turning these two genes into strong candidates to be intimately related to PROT%. On the BTA20 chromosome, *PAIP1*, *C20H5orf34*, *TMEM267*, *CCL28*, *HMGCS1*, and *NIM1K* are the most significant genes located in this genomic region. Of those, only *CCL28* was previously reported to be associated with PROT% in North American Holstein cattle [62]. However, the other five genes also deserve special attention, not only for being highly associated with PROT% but also for being grouped in a narrow genomic region (31.20–31.50 Mb) that possibly makes these genes act together in the expression of PROT%. The C-C motif chemokine ligand 28 gene (*CCL28*) belongs to the subfamily of small cytokine CC genes that are involved in immunoregulatory and inflammatory processes [109]. Because of its relation with antimicrobial activity, *CCL28* can play an important role in mastitis control and thus, indirectly influencing milk production [110]. This gene seems to be a strong candidate gene, as it was identified in our VEP analysis and is directly associated with one of the markers with one of the highest significance levels for PROT% on the BTA20.

Eighteen GO terms were significantly enriched (Table 5) and associated with various processes, especially GO:0005149 (interleukin-1 receptor binding), GO:1903496 (response to 11-deoxycorticosterone), GO:1903494 (response to dehydroepiandrosterone), and GO:0007595 (lactation). As found for FAT%, the interleukin-1 receptor binding presents a large group of IL family genes for PROT%, which are crucial in the expression of milk production traits in many species, including dairy cattle [97]. The terms GO:1903496 and GO:1903494 were also found for PROT, reinforcing the relevance of the casein gene cluster for milk protein, in both forms, total yield and percentage. Additionally, in both terms, the gene *LALBA* (milk whey protein α-lactoalbumin) was also identified for PROT%, highlighting the involvement of this well-known gene with PROT%, as previously reported in other dairy cattle studies [111,112]. On GO:0007595, many genes known for its association with PROT% were identified such as *STAT5A*, *STAT5B*, *ATP2B2*, *CSN3*, *CSN2*, and *PRLR*, validating the connection of these genes with milk composition in dairy cattle. *VDR* has also been previously linked to PROT% in Holstein and Jersey cows [113].

### 4.7. Common Genes Identified in GO Terms

Several overlapping genomic regions were found either between milk production traits or between milk production and LP traits (Figure 2). This fact supports the hypothesis that many genes associated with these traits could have a pleiotropic effect in dairy cattle [16]. According to Oliveira et al. [16], the pleiotropic effects observed on genes related to milk traits suggest a biological function on the use of energy resources directly affecting the synthesis of milk and solids. Few genes have been reported to commonly influence LP and milk production traits, which reinforces the fact that the common genes found in our study can help to elucidate the molecular interactions among various candidate genes with potential pleiotropic effects.

Fifteen genes were significantly associated with LP and at least one of the milk production traits, as *CXCL13* and *LDB2* (MILK); *ZMIZ1*, *bta-mir-371*, *NLRP12* and *PPIF* (FAT); *INPP5A* (FAT%); *SERPINB6*, *SERPINB9*, *IGF2BP1* and *DLX4* (PROT%). Additionally, LP, FAT%, and PROT% had common candidate genes between the three traits simultaneously: *ABI3*, *GNGT2*, *B4GALNT2*, and *PHOSPHO1,* demonstrating their importance on the genetic structure of milk solid production, but also in the duration of peak lactation. Out of those mentioned genes, *SERPINB6* and *SERPINB9* have been previously associated with somatic cell count in Jersey cattle [16] and milk production traits in water buffalo [114]. Furthermore, it is noteworthy that the genes *LDB2* (MILK), *SERPINB6*, *SERPINB9* (PROT%) and *ABI3* (FAT% and PROT%) were also present in the significant GO terms of biological process, revealing its relationship with regulation of cell migration, and regulation of protein and enzyme inhibitor activity.

As expected, due to the high genetic correlation between the studied traits, numerous genes were simultaneously associated with the five milk production traits. Oliveira Junior et al. [13] working with the same North American Holstein population found high genetic correlation (>0.48) among MY, FAT, PROT, FAT%, and PROT% highlighting the close genetic relationship between these traits. Even in multibreed dairy populations the high correlations among production traits are usually observed [115]. In total, 98 common genes were identified for all milk production traits (Figure 2). Interestingly, all these genes are located on BTA14, reinforcing the importance of this genomic region for milk production traits. The genes located closer to the genomic region linked to the top SNP found among MILK, FAT, FAT%, and PROT% are *PPP1R16A*, *FOXH1*, *KIFC2*, and *CYHR1*. Nayeri et al. [15] reported that *PPP1R16A*, *FOXH1*, and *CYHR1* were commonly linked with FAT and FAT% in Holstein cattle and Cai et al. [93] also demonstrated that *PPP1R16A*, *FOXH1*, *KIFC2*, and *CYHR1* presented a potential pleiotropic effect on MILK, FAT, and PROT. Finally, the closer common genes related to the top SNP found for PROT are *GRINA*, *PARP10*, and *PLEC*. *GRINA* and *PARP10* were also reported by Cai et al. [93] as genes with pleiotropic effect on MILK, FAT, and PROT. Furthermore, *PLEC* was the only gene reported to be commonly associated with MILK, PROT, and FAT in Chinese Holstein cattle [17], which is in agreement with our findings.

### 4.8. Potential Implications and Limitations

Several novel candidate genes associated with LP and milk production traits in dairy cattle were identified, while previous associations were also confirmed. These findings will be useful for optimizing genomic prediction of breeding values in Holstein cattle and other dairy breeds, by adding the significant SNPs into commercial SNP panels to increase the accuracy of predictions and also give differential weight to these important genomic regions through biology-driven genomic prediction methods [116]. Furthermore, the genomic regions revealed are initial targets for future studies investigating the molecular mechanisms influencing the phenotypic expression of milk related traits. For instance, some important candidate genes found require a better understanding of their molecular functions, such as *SAE1*, *SERPINB6*, and *SERPINB9*, which were highly associated with LP but their biological functions are not clear. *SAE1*-SUMO activating enzyme subunit 1, is a gene linked to one of the most significant SNPs for LP and also present in one of the enriched GO terms found here, was previously associated with dairy cow mammary gland epithelial cells [117], but its molecular functions related to LP are still unclear.

Additionally, even with an application of a strict quality control to reduce the influence of the poorly imputed variants and individuals on the GWAS analysis, it is still expected that some removed low-frequency variants could be associated with the studied traits and not identified here. Despite the great advantage of identifying causal mutations at low frequency [20], they could also be false positives. Future studies should focus on the biological validation of the key candidate genes reported in this study. This could be done based on in-vitro experiments and gene knock-out models.

## 5. Conclusions

We have shown that the use of imputed whole-genome sequence data for GWAS enabled the identification of a high number of SNPs associated with lactation persistency and milk production traits in dairy cattle. Several genomic regions and candidate genes were identified, which are widely distributed across all autosomal chromosomes, especially BTA5, BTA6, BTA14, BTA18, BTA20, and BTA28. This study also confirmed the importance of the BTA14 for milk production traits. Additionally, many genomic regions with potential pleiotropic effects were identified. Numerous novel candidate genes were revealed: *ARHGAP35*, *NPAS1*, *TMEM160*, *ZC3H4*, *SAE1*, *ZMIZ1*, *PPIF,* *LDB2*, *ABI3*, *SERPINB6*, and *SERPINB9* (LP); *NIM1K*, *ZNF131*, *GABRG1*, *GABRA2*, *DCHS1*, and *SPIDR* (MILK); *NR6A1*, *OLFML2A*, *EXT2*, *POLD1*, *GOT1*, and *ETV6* (FAT); *DPP6*, *LRRC26*, and KCN gene family (FAT%); *CDC14A*, *RTCA*, *HSTN*, and *ODAM* (PROT); *HERC3*, *HERC5*, *LALBA*, *CCL28*, and *NEURL1* (PROT%), involved in key biological pathways such as fatty acid homeostasis, transporter regulator activity, response to progesterone and estradiol, response to steroid hormones, and lactation. Lastly, another relevant finding was that a variety of genomic regions related to LP and milk production were previously associated with fertility traits in dairy cattle, confirming the links between these two groups of traits. Our findings contribute to a better understanding of the molecular mechanisms underlying the phenotypic expression of lactation persistency and milk production traits, which can be useful for improving the genomic evaluation of important economic traits in the Holstein cattle.

## Figures and Tables

**Table 1:** Summary of the pseudo-phenotypes (de-regressed estimated breeding values, dEBVs) for milk (MILK), fat yield (FAT), protein yield (PROT), fat percentage (FAT%), protein percentage (PROT%), and lactation persistency (LP) in North American Holstein cattle.

Traits	Sample Size	dEBVs				Reliability	
		Mean	Min	Max	SD	Mean	SD
MILK	8264	−1507.99	−58,315.8	89,084.9	13,218.18	63.3	9.5
FAT	8262	−57.62	−3416.98	5254.22	893.60	64.7	9.9
PROT	8263	−55.87	−2124.84	2479.37	526.11	60.2	8.9
FAT%	8262	0.27	−37.47	40.01	9.61	64.7	9.9
PROT%	8263	0.11	−17.91	17.96	4.86	60.2	8.9
LP	3447	95.68	−810.60	1008.61	486.37	36.7	6.8

Min: minimum; Max: maximum; SD: standard deviation.

**Table 2:** Description of SNPs, most significant genes, and number of QTLs significantly associated with milk yield (MILK), fat yield (FAT), and fat percentage (FAT%) in North American Holstein cattle.

TRAIT	N ^1^	Chr ^2^	Position (bp)	*p*-Value	Genes (Within ±100 Kb)	QTL ^3^	QTL_Type ^4^
MILK	8	1	141,861,836	2.69 × 10^−6^	*ENSBTAG00000050235, ENSBTAG00000045771, ENSBTAG00000046015*	1	Meat/Carcass
MILK	2	2	80,496,336	9.86 × 10^−6^	*CAVIN2*	0	-
MILK	6	3	26,833,328	2.52 × 10^−6^	*CD58, ATP1A1*	3	Reproduction
MILK	16	4	116,598,211	2.28 × 10^−5^	*DPP6*	4	Milk
MILK	871	5	93,545,576	3.62 × 10^−12^	*MGST1, SLC15A5*	357	Milk
MILK	579	6	86,348,368	2.27 × 10^−10^	*MOB1B, DCK, SLC4A4*	69	Milk
MILK	28	7	5,452,360	7.17 × 10^−6^	*B3GNT3, FCHO1, MAP1S, UNC13A, COLGALT1*	10	Reproduction
MILK	24	8	36,048,320	1.30 × 10^−7^	*bta-mir-2285bg, PTPRD*	1	Meat/Carcass
MILK	91	9	61,950,514	1.39 × 10^−7^	*SPACA1*	0	-
MILK	20	10	87,491,769	5.67 × 10^−8^	*GPATCH2L*	1	Reproduction
MILK	769	11	103,587,292	5.24 × 10^−8^	*NACC2, TMEM250, LHX3*	4	Milk
MILK	1	12	72,628,679	1.02 × 10^−5^	*ENSBTAG00000023309*	0	-
MILK	37	13	6,976,693	2.95 × 10^−10^	*ISM1, TASP1*	15	Exterior
MILK	5564	14	465,742	5.98 × 10^−196^	*ARHGAP39, bta-mir-2308, C14H8orf82, LRRC24, LRRC14, RECQL4, MFSD3, GPT, PPP1R16A, FOXH1, KIFC2, CYHR1, TONSL, VPS28, ENSBTAG00000053637, SLC39A4, CPSF1, ADCK5*	765	Milk
MILK	1012	15	54,030,861	2.13 × 10^−9^	*POLD3, CHRDL2, RNF169, U6, ENSBTAG00000054207, ENSBTAG00000042319*	3	Milk
MILK	308	16	64,637,424	9.60 × 10^−9^	*SMG7, NCF2, ARPC5, APOBEC4*	1	Health
MILK	35	18	56,457,916	5.07 × 10^−6^	*IZUMO2, ENSBTAG00000053322, MYH14, KCNC3, NAPSA, ENSBTAG00000048283, NR1H2, POLD1*	37	Milk
MILK	132	19	8,625,414	1.77 × 10^−6^	*CCDC182, ENSBTAG00000045351, ENSBTAG00000051336, MRPS23, CUEDC1*	15	Reproduction
MILK	1867	20	31,609,872	3.36 × 10^−32^	*NIM1K, ENSBTAG00000042376, ZNF131, ENSBTAG00000054352, ENSBTAG00000052195, ENSBTAG00000051111*	27	Milk
MILK	27	21	33,151,073	6.13 × 10^−6^	*ENSBTAG00000051111, ODF3L1, CSPG4, SNX33*	2	Health
MILK	6	22	21,859,149	4.59 × 10^−6^	*bta-mir-2285am, U6, SUMF1*	0	-
MILK	436	23	12,589,558	6.37 × 10^−8^	*GLO1, DNAH8*	0	-
MILK	6	24	21,019,665	7.60 × 10^−6^	*MOCOS, ELP2, SLC39A6, RPRD1A*	0	-
MILK	40	28	18,592,444	1.03 × 10^−6^	*ZNF365*	5	Milk
MILK	16	29	9,512,241	6.55 × 10^−6^	*PICALM*	9	Milk
FAT	30	1	126,143,578	8.14 × 10^−8^	*ENSBTAG00000038111, PCOLCE2*	3	Reproduction
FAT	46	2	67,353,179	3.19 × 10^−6^	*DPP10, ENSBTAG00000050341,*	0	-
FAT	15	4	28,468,986	5.19 × 10^−8^	*POLR1F, ENSBTAG00000050341, TMEM196*	0	-
FAT	1580	5	93,627,511	8.00 × 10^−26^	*SLC15A5*	167	Milk
FAT	31	6	36,205,216	1.00 × 10^−7^	*HERC3, PIGY, HERC5*	202	Milk
FAT	18	7	21,215,200	5.74 × 10^−6^	*GADD45B, LMNB2, TIMM13, TMPRSS9, SPPL2B, LSM7, LINGO3, PEAK3, OAZ1*	23	Reproduction
FAT	8	8	42,176,903	6.89 × 10^−6^	*ENSBTAG00000051041*	7	Milk
FAT	26	10	86,711,416	4.16 × 10^−6^	*JDP2, bta-mir-10162, BATF, FLVCR2*	3	Health
FAT	1275	11	95,801,995	2.86 × 10^−9^	*NR6A1, bta-mir-181a-2, bta-mir-181b-2, OLFML2A, U6, WDR38, RPL35, ARPC5L, GOLGA1*	1	Production
FAT	12	12	69,122,129	1.67 × 10^−5^	*ENSBTAG00000051519, 5S_rRNA, TGDS, GPR180, U2, SOX21*	1	Exterior
FAT	322	13	54,614,993	1.43 × 10^−7^	*DIDO1, TCFL5, COL9A3, OGFR, MRGBP, NTSR1, SLCO4A1, ENSBTAG00000051754, ENSBTAG00000051012*	3	Milk
FAT	4490	14	465,742	2.08 × 10^−183^	*ARHGAP39, bta-mir-2308, C14H8orf82, LRRC24, LRRC14, RECQL4, MFSD3, GPT, PPP1R16A, FOXH1, KIFC2, CYHR1, TONSL, VPS28, ENSBTAG00000053637, SLC39A4, CPSF1, ADCK5*	765	Milk
FAT	181	15	74,165,872	1.09 × 10^−9^	*ACCSL, ACCS, EXT2*	6	Reproduction
FAT	260	16	61,287,081	9.53 × 10^−9^	*CEP350, QSOX1*	0	-
FAT	12	18	60,972,942	2.06 × 10^−5^	*bta-mir-371, NLRP12, MGC157082, ENSBTAG00000014953*	50	Milk
FAT	35	19	34,952,167	1.85 × 10^−6^	*NT5M, COPS3, FLCN, PLD6, MPRIP*	7	Production
FAT	18	22	27,251,453	4.27 × 10^−6^	*CNTN3*	3	Health
FAT	13	23	11,102,541	3.20 × 10^−6^	*ENSBTAG00000048838, PIM1, ENSBTAG00000045936, TMEM217, TBC1D22B*	0	-
FAT	639	26	21,354,112	1.07 × 10^−9^	*PKD2L1, SCD, bta-mir-12016, WNT8B, SEC31B, NDUFB8, HIF1AN*	296	Milk
FAT	6	28	34,969,442	7.51 × 10^−6^	*ZMIZ1, PPIF, ZCCHC24*	2	Production
FAT	20	29	49,353,940	1.30 × 10^−6^	*TSPAN32, ASCL2, TH, INS, IGF2*	8	Milk
FAT%	8	1	1,025,407	1.89 × 10^−5^	*RCAN1, KCNE1, ENSBTAG00000026259, ENSBTAG00000051226, FAM243A, SMIM11A, KCNE2*	6	Milk
FAT%	47	2	128,607,054	8.79 × 10^−7^	*STPG1, GRHL3, U6*	2	Milk
FAT%	29	4	106,456,105	6.10 × 10^−6^	*ENSBTAG00000049510, ENSBTAG00000048380, ENSBTAG00000053286, OR6V1, ENSBTAG00000052365, PIP, ENSBTAG00000050494*	0	-
FAT%	2070	5	93,627,511	3.55 × 10^−34^	*SLC15A5*	167	Milk
FAT%	41	6	36,205,216	5.31 × 10^−12^	*HERC3, PIGY, HERC5*	202	Milk
FAT%	19	7	21,215,200	2.60 × 10^−5^	*GADD45B, LMNB2, TIMM13, TMPRSS9, SPPL2B, LSM7, LINGO3, PEAK3, OAZ1*	23	Reproduction
FAT%	11	8	88,461,261	8.40 × 10^−8^	*GADD45G*	10	Reproduction
FAT%	20	9	61,950,514	9.91 × 10^−7^	*SPACA1*	0	-
FAT%	3	10	77,587,306	4.17 × 10^−6^	*FUT8*	4	Meat/Carcass
FAT%	2365	11	105,500,024	6.21 × 10^−6^	*COL5A1, FCN1, ENSBTAG00000054425, OLFM1, ENSBTAG00000052600*	15	Milk
FAT%	6	12	70,194,006	2.47 × 10^−6^	*ENSBTAG00000047383*	0	-
FAT%	161	13	47,813,637	3.64 × 10^−7^	*GPCPD1, ENSBTAG00000054005, ENSBTAG00000051557*	7	Milk
FAT%	6274	14	465,742	2.82 × 10^−317^	*ARHGAP39, bta-mir-2308, C14H8orf82, LRRC24, LRRC14, RECQL4, MFSD3, GPT, PPP1R16A, FOXH1, KIFC2, CYHR1, TONSL, VPS28, ENSBTAG00000053637, SLC39A4, CPSF1, ADCK5*	765	Milk
FAT%	789	15	51,993,618	9.14 × 10^−9^	*CLPB, PDE2A, ENSBTAG00000050827*	2	Production
FAT%	714	16	61,059,994	7.32 × 10^−10^	*FAM163A, TOR1AIP2, TOR1AIP1, U6*	11	Reproduction
FAT%	33	17	65,230,489	1.97 × 10^−6^	*KIAA1671, 7SK, ENSBTAG00000053952, CRYBB3, CRYBB2*	18	Reproduction
FAT%	215	19	35,457,767	2.41 × 10^−7^	*KCNJ12, UTP18, MBTD1*	0	-
FAT%	1293	20	29,991,518	2.95 × 10^−23^	*ENSBTAG00000054476, MRPS30*	179	Milk
FAT%	6	25	11,488,475	4.73 × 10^−6^	*CPPED1*	0	-
FAT%	8	26	51,156,653	5.45 × 10^−6^	*INPP5A, ENSBTAG00000051139, BNIP3, ENSBTAG00000050527*	2	Reproduction
FAT%	3	27	36,400,257	1.07 × 10^−5^	*5S_rRNA, GOLGA7, GINS4*	27	Milk
FAT%	13	28	26,978,779	1.00 × 10^−5^	*ADAMTS14, TBATA, SGPL1, ENSBTAG00000054819, PCBD1*	18	Milk

^1^ Number of genes present in each chromosome; ^2^ Chr = chromosome; ^3^ QTL = number of QTL previously reported in Animal QTLdb; ^4^ QTL_type = main type of QTL trait group previously identified.

**Table 3:** Description of SNPs, most significant genes, and number of QTLs significantly associated with protein yield (PROT), protein percentage (PROT%), and lactation persistency (LP) in North American Holstein cattle.

TRAIT	N ^1^	Chr ^2^	Position (bp)	*p*-Value	Genes (Within ±100 Kb)	QTL ^3^	QTL_Type ^4^
PROT	236	1	117,208,394	1.39 × 10^−7^	*CLRN1*	1	Reproduction
PROT	12	2	1,465,207	8.33 × 10^−6^	*AMER3*	3	Reproduction
PROT	16	3	42,939,326	8.31 × 10^−8^	*CDC14A, ENSBTAG00000054319, ENSBTAG00000015759, RTCA*	0	-
PROT	61	4	106,606,419	8.84 × 10^−6^	*ENSBTAG00000050494, TAS2R39, TAS2R40, GSTK1, TMEM139, CASP2, CLCN1*	0	-
PROT	72	5	91,526,305	5.46 × 10^−6^	*PIK3C2G, ENSBTAG00000046178*	23	Milk
PROT	65	6	86,795,218	4.49 × 10^−7^	*SLC4A4*	278	Milk
PROT	6	7	93,911,428	1.49 × 10^−5^	*KIAA0825, SLF1*	0	-
PROT	30	8	948,000	6.96 × 10^−7^	*PALLD, 5S_rRNA*	16	Production
PROT	21	10	10,774,713	1.37 × 10^−6^	*CMYA5, SNORA72, ENSBTAG00000049054*	0	-
PROT	121	11	77,730,244	1.57 × 10^−7^	*TDRD15*	4	Production
PROT	8	12	77,353,297	8.04 × 10^−6^	*TMTC4, ENSBTAG00000053717*	0	-
PROT	489	13	33,556,356	2.02 × 10^−9^	*ARHGAP12*	5	Reproduction
PROT	1541	14	827,938	3.24 × 10^−22^	*MAF1, ENSBTAG00000051469, SHARPIN, CYC1, GPAA1, EXOSC4, OPLAH, ENSBTAG00000015040, SPATC1, GRINA, PARP10, PLEC, bta-mir-2309*	670	Milk
PROT	32	15	41,254,106	5.23 × 10^−7^	*GALNT18*	1	Milk
PROT	22	17	65,001,641	5.01 × 10^−6^	*ENSBTAG00000054184, PIWIL3, SGSM1, TMEM211*	3	Reproduction
PROT	48	18	36,657,423	1.48 × 10^−6^	*CYB5B, ENSBTAG00000052086, NFAT5*	0	-
PROT	37	19	42,052,275	6.05 × 10^−6^	*JUP, P3H4, FKBP10, NT5C3B, KLHL10, KLHL11, ACLY, ENSBTAG00000050335, TTC25, CNP, DNAJC7*	2	Milk
PROT	7	21	10,379,606	2.70 × 10^−6^	*ENSBTAG00000049351*	8	Reproduction
PROT	30	22	23,112,217	3.53 × 10^−6^	*CRBN, TRNT1, IL5RA*	4	Milk
PROT	628	23	5,551,604	3.67 × 10^−7^	*FAM83B*	2	Milk
PROT	31	24	21,507,281	1.32 × 10^−6^	*GALNT1, INO80C*	0	-
PROT	9	25	10,257,579	5.43 × 10^−6^	*ENSBTAG00000050716, ENSBTAG00000050363, LITAF*	2	Milk
PROT	8	26	15,946,065	5.94 × 10^−6^	*PLCE1, NOC3L, U6, TBC1D12, ENSBTAG00000051299, ENSBTAG00000049089, HELLS, 7SK*	13	Milk
PROT	15	28	1,284,944	1.04 × 10^−6^	*RAB4A, CCSAP, ENSBTAG00000048654, ENSBTAG00000050985*	7	Milk
PROT%	56	1	142,907,564	3.41 × 10^−6^	*SLC37A1, PDE9A*	98	Milk
PROT%	106	3	9,965,020	5.49 × 10^−10^	*FCRL6, DUSP23, CRP*	9	Milk
PROT%	39	4	9,165,220	3.80 × 10^−6^	*ENSBTAG00000052341, MTERF1*	0	-
PROT%	828	5	93,655,680	1.81 × 10^−12^	*SLC15A5*	172	Milk
PROT%	696	6	36,205,216	1.28 × 10^−18^	*HERC3, PIGY, HERC5*	202	Production
PROT%	6	7	104,076,138	1.10 × 10^−5^	*U6*	0	-
PROT%	16	8	1,868,612	2.86 × 10^−6^	*MFAP3L, ENSBTAG00000051098, AADAT*	6	Reproduction
PROT%	61	9	26,103,917	4.56 × 10^−8^	*TPD52L1, RNF217*	0	-
PROT%	36	10	40,647,347	2.08 × 10^−6^	*ENSBTAG00000034580*	3	Reproduction
PROT%	1309	11	63,476,482	1.52 × 10^−10^	*SLC1A4, CEP68, RAB1A*	26	Milk
PROT%	5	12	76,184,908	2.08 × 10^−5^	*UBAC2, GPR18, GPR183, ENSBTAG00000038268*	4	Milk
PROT%	100	13	46,366,498	2.96 × 10^−7^	*ADARB2, ENSBTAG00000054346, WDR37, IDI1, GTPBP4, U6, LARP4B, ENSBTAG00000051962*	15	Milk
PROT%	5949	14	465,742	2.75 × 10^−122^	*ARHGAP39, bta-mir-2308, C14H8orf82, LRRC24, LRRC14, RECQL4, MFSD3, GPT, PPP1R16A, FOXH1, KIFC2, CYHR1, TONSL, VPS28, ENSBTAG00000053637, SLC39A4, CPSF1, ADCK5*	765	Milk
PROT%	2221	15	51,232,796	1.85 × 10^−13^	*STIM1, RHOG, PGAP2, NUP98*	14	Health
PROT%	749	16	60,724,655	2.34 × 10^−9^	*SOAT1, AXDND1, NPHS2, TDRD5*	4	Milk
PROT%	997	19	35,457,767	4.58 × 10^−9^	*KCNJ12, UTP18, MBTD1*	0	-
PROT%	2201	20	31,391,058	5.47 × 10^−45^	*PAIP1, ENSBTAG00000049623, C20H5orf34, TMEM267, CCL28, HMGCS1, ENSBTAG00000048672, NIM1K*	72	Milk
PROT%	40	22	54,244,267	3.21 × 10^−6^	*CLEC3B, EXOSC7, ZDHHC3, TMEM42, GHRL, SEC13*	3	Milk
PROT%	554	23	47,176,195	8.66 × 10^−10^	*SLC35B3*	0	-
PROT%	25	24	56,331,031	3.77 × 10^−6^	*WDR7*	0	-
PROT%	5	25	14,923,140	2.20 × 10^−6^	*ENSBTAG00000051040*	6	Milk
PROT%	281	26	23,088,324	2.56 × 10^−8^	*GBF1, NFKB2, PSD, FBXL15, CUEDC2, bta-mir-146b, MFSD13A, ACTR1A, SUFU*	40	Milk
PROT%	26	28	35,624,139	1.40 × 10^−5^	*ENSBTAG00000048082, SFTPD, MBL1, SFTPA1, ENSBTAG00000052322, MAT1A, DYDC1*	2	Health
PROT%	686	29	40,803,159	4.78 x10^−10^	*ASRGL1, ENSBTAG00000042287, SCGB1A1, AHNAK*	19	Milk
LP	2	4	19,848,832	1.74 × 10^−6^	*THSD7A*	2	Milk
LP	23	6	104,139,800	6.86 × 10^−6^	*STK32B, 5S_rRNA, CYTL1*	8	Milk
LP	3	7	2,852,946	9.20 × 10^−6^	*ENSBTAG00000051744, ENSBTAG00000052719, ENSBTAG00000049190*	1	Milk
LP	3	8	59,108,254	1.81 × 10^−6^	*ENSBTAG00000042498, ENSBTAG00000049991, FAM205C*	0	-
LP	2	9	11,532,901	2.65 × 10^−5^	*RIMS1*	1	Reproduction
LP	5	12	68,955,769	1.48 × 10^−5^	*ENSBTAG00000054671, ENSBTAG00000051263, DCT, ENSBTAG00000051519, 5S_rRNA*	8	Milk
LP	0	14	10,086,164	9.93 × 10^−6^	*-*	17	Milk
LP	2	15	17,222,016	1.88 × 10^−5^	*ELMOD1, SLN*	3	Reproduction
LP	0	17	35,605,687	1.69 × 10^−5^	*-*	0	-
LP	26	18	54,117,753	8.56 × 10^−7^	*ARHGAP35, NPAS1, TMEM160, ZC3H4, SAE1*	4	Production
LP	25	19	36,768,672	5.36 × 10^−6^	*DLX4, ENSBTAG00000045805, U6, ENSBTAG00000053450, ENSBTAG00000049677, KAT7, ENSBTAG00000052793*	20	Milk
LP	4	21	31,125,876	2.14 × 10^−5^	*UBE2Q2, ENSBTAG00000048528, FBXO22, ENSBTAG00000043187*	0	-
LP	3	22	48,814,749	1.19 × 10^−6^	*POC1A, DUSP7*	4	Milk
LP	16	23	15,502,651	1.39 × 10^−5^	*FOXP4, MDFI, TFEB, PGC, FRS3, ENSBTAG00000038916, TOMM6*	3	Milk
LP	15	26	51,053,430	5.16 × 10^−6^	*INPP5A, ENSBTAG00000054967*	0	-
LP	2	27	35,948,511	3.46 × 10^−5^	*ZMAT4*	1	Meat/Carcass
LP	2	28	34,869,857	5.28 × 10^−7^	*ZMIZ1, PPIF*	5	Milk
LP	1	29	22,382,652	2.17 × 10^−5^	*SLC17A6*	0	-

^1^ Number of genes identified in each chromosome; ^2^ Chr = chromosome; ^3^ QTL = number of previously QTL reported in Animal QTLdb; ^4^ QTL_type = main type of QTL trait group previously identified.(a).

**Table 4:** Most significantly enriched gene ontology (GO) terms of candidate genes for milk yield (MILK), fat yield (FAT), and fat percentage (FAT%) in North American Holstein cattle.

Trait	GO	Term	*p*-Value	Genes
MILK	GO:0004890	GABA-A receptor activity	2.4 × 10^−4^	*GABRA2, GABRG1, GABRA4, GABRB1, and GABRD*
MILK	GO:0006749	Glutathione metabolic process	6.7 × 10^−4^	*OPLAH, ALDH5A1, CLIC5, GSTA2, GSTA3, GSTA4, GSTK1, and MGST1*
MILK	GO:0005230	Extracellular ligand-gated ion channel activity	7.9 × 10^−4^	*GABRA2, GABRG1, GABRA4, GABRB1, and GABRD*
MILK	GO:1903496	Response to 11-deoxycorticosterone	1.4 × 10^−3^	*CSN1S1, CSN1S2, CSN2, and CSN3*
MILK	GO:0007605	sensory perception of sound	3.2 × 10^−3^	*BARHL1, EYA4, FBXO11, NIPBL, USH1G, CLIC5, CHD7, COL2A1, DCDC2, MYH14, SNAI2, SLC1A3, and TUB*
MILK	GO:0005513	Detection of calcium ion	7.0 × 10^−3^	*CALM2, CALM3, KCNMB4, and STIM1*
MILK	GO:0043950	Positive regulation of cAMP-mediated signaling	7.0 × 10^−3^	*CXCL10, CXCL11, CXCL9, and PTGIR*
MILK	GO:0003273	Cell migration involved in endocardial cushion formation	8.3 × 10^−3^	*DCHS1, NOTCH1, and SNAI2*
MILK	GO:0071479	Cellular response to ionizing radiation	9.4 × 10^−3^	*FBXO4, RAD1, CLOCK, EEF1D, SPIDR, and SNAI2*
MILK	GO:0004364	Glutathione transferase activity	1.0 × 10^−3^	*CLIC5, GSTA2, GSTA3, GSTA4, GSTA5, GSTK1, and MGST1*
FAT	GO:0015125	Bile acid transmembrane transporter activity	4.1 × 10^−4^	*SLCO1A2, SLCO1B3, SLCO1C1,* and *SLCO2B1*
FAT	GO:0055089	Fatty acid homeostasis	5.4 × 10^−4^	*POLD1, DGAT1, GOT1, GPAM,* and *INS*
FAT	GO:0030154	Cell differentiation	6.1 × 10^−4^	*DHCR7, EHF, ETV6, EYA3, HCK, SPIB, CREBL2, EXT2, GADD45B, GADD45G, MGP, NR5A1, PPDPF, PRRC2B, PTK2, PTK6, RGS19, SCX, SFRP5, STYK1, SNAPC4, SRMS, TRAPPC9, and TTF1*
FAT	GO:0007275	Multicellular organism development	2.4 × 10^−3^	*ALX4, DDX1, EYA3, SUFU, TNFRSF1A, TNFRSF6B, GADD45B, GADD45G, LBH, LTBR, PPDPF, PLCZ1, SFRP5, STRBP, SPRED2, TPI1, TRIM5,4 and ZFAT*
FAT	GO:0000978	RNA polymerase II core promoter proximal region sequence-specific DNA binding	2.7 × 10^−3^	*AEBP2, EHF, ETV6, FEZF2, FOSL2, JDP2, MAFA, MXD1, MEIS1, NACC2, SOX18, TLX1, ASCL2, BHLHE41, BATF, CHD7, FOXJ2, GMEB2, HSF1, HHEX, NR1H2, NR6A1, OTX1, TP73, and ZGPAT*
FAT	GO:0042127	Regulation of cell proliferation	4.3 × 10^−3^	*DHCR7, HCK, NDRG1, NKX2-3, SRC, TNFRSF1A, TNFRSF6B, APOBEC1, GUCY2C, HHEX, LTBR, PTGS1, PTK2, PTK6, STYK1, and SRMS*
FAT	GO:0016509	Long-chain-3-hydroxyacyl-CoA dehydrogenase activity	6.5 × 10^−3^	*HADHA, HADHB, and HSD17B12*
FAT	GO:0036094	Small molecule binding	7.2 × 10^−3^	*LCN2, LCN9, PAEP, and RBP4*
FAT	GO:0001671	ATPase activator activity	9.9 × 10^−3^	*AHSA2, ATP1B3, TOR1AIP1, and TOR1AIP2*
FAT%	GO:0005149	Interleukin-1 receptor binding	8.5 × 10^−8^	*IL1A, IL1B, IL1F10, IL1RN, IL36RN, IL36A, IL36B, IL36G, and IL37*
FAT%	GO:0007585	Respiratory gaseous exchange	7.6 × 10^−4^	*PBX3, TLX3, CHST11, FUT8, GRIN1, SFTPB, and SFTPD*
FAT%	GO:0015125	Bile acid transmembrane transporter activity	9.1 × 10^−4^	*SLCO1A2, SLCO1B3, SLCO1C1, and SLCO2B1*
FAT%	GO:0015459	Potassium channel regulator activity	3.6 × 10^−3^	*DPP6, LRRC26, KCNMB4, KCNIP4, KCNE1, KCNE2, and KCNE3*
FAT%	GO:0005031	Tumor necrosis factor-activated receptor activity	5.1 × 10^−3^	*RELT, TNFRSF1A, TNFRSF1B, TNFRSF8, LTBR, and NGFR*

**Table 5:** Most significantly enriched gene ontology (GO) terms of candidate genes for protein yield (PROT), protein percentage (PROT%), and lactation persistency (LP) in North American Holstein cattle.

Trait	GO	Term	*p*-Value	Genes
PROT	GO:0008289	Lipid binding	1.7 × 10^−5^	*BPIFA1, BPIFA3, BPIFA2A, BPIFA2B, BPIFA2C, BPIFB1, BPIFB2, BPIFB3, BPIFB4, BPIFB6, ACBD7, and PLTP*
PROT	GO:0016998	Cell wall macromolecule catabolic process	6.9 × 10^−5^	*LYSB, LYZ1, LYZ3, LYZ2, and LYZ*
PROT	GO:0003796	Lysozyme activity	8.0 × 10^−5^	*LYSB, LYZ1, LYZ3, LYZ2, and LYZ*
PROT	GO:0042742	Defense response to bacterium	1.2 × 10^−4^	*DEFB122, DEFB122A, CSN1S2, DEFB116, DEFB119, DEFB123, DEFB124, DEFB119, HSTN, LYZ1, and NOD2*
PROT	GO:0019835	Cytolysis	1.7 × 10^−4^	*LYSB, LYZ1, LYZ3, LYZ2, and LYZ*
PROT	GO:1903496	Response to 11-deoxycorticosterone	2.4 × 10^−4^	*CSN1S1, CSN1S2, CSN2, and CSN3*
PROT	GO:0050829	Defense response to Gram-negative bacterium	8.5 × 10^−4^	*BPI, LYSB, LYZ1, LYZ3, LYZ2, and LYZ*
PROT	GO:0045087	Innate immune response	2.7 × 10^−3^	*BPIFA1, BPIFB1, BPIFB3, CYLD, HCK, DEFB122, DEFB122A, DEFB116, DEFB119, DEFB123, DEFB124, NOD2, TRIM10, TRIM15, and TRIM31*
PROT	GO:0032570	Response to progesterone	3.4 × 10^−3^	*CSN1S1, CSN1S2, CSN2, and CSN3*
PROT	GO:0032355	Response to estradiol	3.4 × 10^−3^	*CSN1S1, CSN1S2, CSN2, and CSN3*
PROT	GO:0007586	Digestion	6.4 × 10^−3^	*LYZ1, LYZ3, LYZ2, and UCN3*
PROT	GO:0045028	G-protein coupled purinergic nucleotide receptor activity	6.4 × 10^−3^	*GPR171, GPR87, P2RY12, and P2RY14*
PROT	GO:0050830	Defense response to Gram-positive bacterium	9.2 × 10^−3^	*H2B, LYSB, LYZ1, LYZ3, LYZ2, and LYZ*
PROT%	GO:0005149	Interleukin-1 receptor binding	9.0 × 10^−8^	*IL1A, IL1RN, IL36A, IL36B, IL37, IL1B, IL36G, IL36RN, IRAK4, and IL1F10*
PROT%	GO:1903496	Response to 11-deoxycorticosterone	3.5 × 10^−4^	*CSN1S2, CSN3, LALBA, CSN1S1, and CSN2*
PROT%	GO:1903494	Response to dehydroepiandrosterone	3.5 × 10^−4^	*CSN1S2, CSN3, LALBA, CSN1S1, and CSN2*
PROT%	GO:0005452	Inorganic anion exchanger activity	3.8 × 10^−4^	*SLC22A12, SLC4A8, SLC22A6, SLC22A8, SLC4A4, SLC22A10, SLC4A5, SLC22A9, and SLC22A11*
PROT%	GO:0032355	Response to estradiol	2.0 × 10^−3^	*STAT3, CSN1S2, CSN3, LALBA, CSN1S1, and CSN2*
PROT%	GO:0015347	Sodium-independent organic anion transmembrane transporter activity	2.2 × 10^−3^	*SLC22A12, SLC22A6, SLCO4A1, SLCO2B1, SLC22A8, SLC22A10, SLC22A9, and SLC22A11*
PROT%	GO:0046983	Protein dimerization activity	2.6 × 10^−3^	*TFAP2A, PPP3CA, STAT5B, HEY1, MYC, ID2, TCF23, STAT3, ANO4, and E2F6*
PROT%	GO:0043252	Sodium-independent organic anion transport	3.0 × 10^−3^	*SLC22A12, SLC22A6, SLCO4A1, SLCO2B1, SLC22A8, SLC22A10, SLC22A9, and SLC22A11*
PROT%	GO:0043153	Entrainment of circadian clock by photoperiod	3.3 × 10^−3^	*PPP1CB, PER1, RBM4, ID2, CRY1, RBM4B, and TP53*
PROT%	GO:0007595	Lactation	3.9 × 10^−3^	*STAT5A, STAT5B, VDR, NEURL1, ATP2B2, CSN3, CSN2, and PRLR*
PROT%	GO:0007259	JAK-STAT cascade	4.8 × 10^−3^	*STAT5A, STAT5B, CTR9, IL31RA, PRLR, and SOCS5*
PROT%	GO:0030282	Bone mineralization	5.1 × 10^−3^	*KLF10, CLEC3B, WNT11, PKDCC, RSPO2, FBXL15, IFITM5, and LGR4*
PROT%	GO:0048013	Ephrin receptor signaling pathway	6.2 × 10^−3^	*EPHB6, EFNA1, EFNA3, EFNB3, NCK2, EFNA4, and PTK2*
PROT%	GO:0010628	Positive regulation of gene expression	6.4 × 10^−3^	*CRP, SEC16B, OSR2, RAMP2, PRKAA1, ODAM, ROCK2, RBM4B, SCX, SERPINB9, LRRC32, WNT11, FGF8, FABP4, ID2, KIT, RPS3, DROSHA, STAP1, KRAS, APOB, IL7R, ZBTB7B, and ZPR1*
PROT%	GO:0008380	RNA splicing	6.5 × 10^−3^	*RBFOX2, MTERF3, PRPF4B, MAGOHB, JMJD6, RBM4, RBMXL2, PUF60, C1QBP, SRSF2, LUC7L3, PABPC1, SRSF7, and ZPR1*
PROT%	GO:0005344	Oxygen transporter activity	6.9 × 10^−3^	*MB, HBE2, HBE1, HBE4, HBB, and CYGB*
PROT%	GO:0006397	mRNA processing	7.1 × 10^−3^	*RNASEL, RBFOX2, DDX1, PRPF4B, HNRNPLL, MAGOHB, JMJD6, RBM4, RBMXL2, ALKBH5, PUF60, C1QBP, SRSF2, PABPC1, AURKAIP1, SRSF7, and ZPR1*
PROT%	GO:0048704	Embryonic skeletal system morphogenesis	7.8 × 10^−3^	*OSR2, COL11A1, PCGF2, HOXB4, HOXB3, HOXB2, HOXB1, HOXB7, and DSCAML1*
LP	GO:0030334	Regulation of cell migration	1.3 × 10^−1^	*ABI3 and LDB2*
LP	GO:1903955	Positive regulation of protein targeting to mitochondrion	1.5 × 10^−1^	*ELMOD1 and SAE1*
LP	GO:0004867	Serine-type endopeptidase inhibitor activity	1.9 × 10^−1^	*SERPINB6 and SERPINB9*

## Data Availability

All the data supporting the results of this study are included in the article and in the Supplementary Materials Section.

## Supplementary Materials

The following are available online at https://www.mdpi.com/article/10.3390/genes12111830/s1, Figure S1: Manhattan plots with truncated *Y*-axis for the GWAS results for milk yield (MILK), fat yield (FAT), fat percentage (FAT%), protein yield (PROT) and protein percentage (PROT%), based on imputed whole-genome sequence data. Figure S2: Gene interaction network for genes associated with lactation persistency in North American Holstein cattle. Table S1: Description of significant SNPs, associated genes id and name, and gene biotype for milk yield (MILK) in North American Holstein cattle. Table S2: Description of significant SNPs, associated genes id and name, and gene biotype for fat yield (FAT) in North American Holstein cattle. Table S3: Description of significant SNPs, associated genes id and name, and gene biotype for fat percentage (FAT%) in North American Holstein cattle. Table S4: Description of significant SNPs, associated genes id and name, and gene biotype for protein yield (PROT) in North American Holstein cattle Table S5: Description of significant SNPs, associated genes id and name, and gene biotype for protein percentage (PROT%) in North American Holstein cattle. Table S6: Variants identified in variant effect predictor analysis for LP, MILK, FAT, FAT%, PROT, and PROT% in North American Holstein cattle. Table S7: Description of significant SNPs, associated genes id and name, and gene biotype for lactation persistency (LP) in North American Holstein cattle.

## References

[1] Han B., Yuan Y., Li Y., Liu L., Sun D. (2019). Single nucleotide polymorphisms of NUCB2 and their genetic associations with milk production. Genes.

[2] Brito L.F., Bedere N., Douhard F., Oliveira H.R., Arnal M., Peñagaricano F., Schinckel A.P., Baes C.F., Miglior F. (2021). Genetic selection of high-yielding dairy cattle toward sustainable farming systems in a rapidly changing world. Animal.

[3] Sehested J., Gaillard C., Lehmann J.O., Maciel G.M., Vestergaard M., Weisbjerg M.R., Mogensen L., Larsen L.B., Poulsen N.A., Kristensen T. (2019). Extended lactation in dairy cattle. Animal.

[4] Gaines W.L. (1927). Persistency of Lactation in Dairy Cows: A Preliminary Study of Certain Guernsey and Holstein Records.

[5] Danell B. (1982). Studies on lactation yield and individual test-day yields of Swedish dairy cows: IV. Extension of part-lactation records for use in sire evaluation. Acta Agric. Scand..

[6] Grossman M., Hartz S.M., Koops W.J. (1999). Persistency of lactation yield: A novel approach. J. Dairy Sci..

[7] Cole J.B., Null D.J. (2009). Genetic evaluation of lactation persistency for five breeds of dairy cattle. J. Dairy Sci..

[8] Dhakal K., Tiezzi F., Clay J.S., Maltecca C. (2016). Causal relationships between clinical mastitis events, milk yields and lactation persistency in US Holsteins. Livest. Sci..

[9] Yamazaki T., Takeda H., Osawa T., Yamaguchi S., Hagiya K. (2019). Genetic correlations among fertility traits and lactation persistency within and across Holstein herds with different milk production during the first three lactations. Livest. Sci..

[10] Loker S., Bastin C., Miglior F. (2012). Genetic and environmental relationships between body condition score and milk production traits in Canadian Holsteins. J. Dairy Sci..

[11] Miglior F., Fleming A., Malchiodi F., Brito L.F., Martin P., Baes C.F. (2017). A 100-year review: Identification and genetic selection of economically important traits in dairy cattle. J. Dairy Sci..

[12] Do D.N., Fleming A., Schenkel F.S., Miglior F., Zhao X., Ibeagha-awemu E.M. (2018). Genetic parameters of milk cholesterol content in Holstein cattle. Can. J. Anim. Sci..

[13] Oliveira G.A., Schenkel F.S., Alcantara L., Houlahan K., Lynch C., Baes C.F. (2021). Estimated genetic parameters for all genetically evaluated traits in Canadian Holsteins. J. Dairy Sci..

[14] Cochran S.D., Cole J.B., Null D.J., Hansen P.J. (2013). Discovery of single nucleotide polymorphisms in candidate genes associated with fertility and production traits in Holstein cattle. BMC Genet..

[15] Nayeri S., Sargolzaei M., Abo-ismail M.K., May N., Miller S.P., Schenkel F., Moore S.S., Stothard P. (2016). Genome-wide association for milk production and female fertility traits in Canadian dairy Holstein cattle. BMC Genet..

[16] Oliveira H.R., Cant J.P., Brito L.F., Feitosa F.L.B., Chud T.C.S., Fonseca P.A.S., Jamrozik J., Silva F.F., Lourenco D.A.L., Schenkel F.S. (2019). Genome-wide association for milk production traits and somatic cell score in different lactation stages of Ayrshire, Holstein, and Jersey dairy cattle. J. Dairy Sci..

[17] Wang D., Ning C., Liu J., Zhang Q., Jiang L. (2019). Association studies for milk production traits in Chinese Holstein by an efficient rotated linear mixed model. J. Dairy Sci..

[18] Van Der Berg I., Boichard D., Lund M.S. (2016). Comparing power and precision of within-breed and multibreed genome-wide association studies of production traits using whole- genome sequence data for 5 French and Danish dairy cattle breeds. J. Dairy Sci..

[19] Larmer S.G., Sargolzaei M., Brito L.F., Ventura R.V., Schenkel F.S. (2017). Novel methods for genotype imputation to whole-genome sequence and a simple linear model to predict imputation accuracy. BMC Genet..

[20] Hayes B.J., Macleod I.M., Daetwyler H.D., Bowman P.J., Chamberlian A.J., Vander Jagt C.J., Capitan A., Pausch H., Stothard P., Liao X. (2014). Genomic prediction from whole genome sequence in livestock: The 1000 bull genomes project. Proceedings of the World Congress of Genetics Applied to Livestock Production.

[21] Chen S.-Y., Oliveira H.R., Schenkel F.S., Pedrosa V.B., Melka M.G., Brito L.F. (2020). Using imputed whole-genome sequence variants to uncover candidate mutations and genes affecting milking speed and temperament in Holstein cattle. J. Dairy Sci..

[22] Moghaddar N., Khansefid M., van der Werf J.H.J., Bolormaa S., Duijvesteijn N., Clark S.A., Swan A.A., Daetwyler H.D., MacLeod I.M. (2019). Genomic prediction based on selected variants from imputed whole-genome sequence data in Australian sheep populations. Genet. Sel. Evol..

[23] Van den Berg S., Vandenplas J., van Eeuwijk F.A., Bouwman A.C., Lopes M.S., Veerkamp R.F. (2019). Imputation to whole-genome sequence using multiple pig populations and its use in genome-wide association studies. Genet. Sel. Evol..

[24] Talouarn E., Bardou P., Palhière I., Oget C., Clément V., Tosser-Klopp G., Rupp R., Robert-Granié C. (2020). Genome wide association analysis on semen volume and milk yield using different strategies of imputation to whole genome sequence in French dairy goats. BMC Genet..

[25] Teissier M., Sanchez M.P., Boussaha M., Barbat A., Hoze C., Robert-Granie C., Croiseau P. (2018). Use of meta-analyses and joint analyses to select variants in whole genome sequences for genomic evaluation: An application in milk production of French dairy cattle breeds. J. Dairy Sci..

[26] Xiang R., van den Berg I., MacLeod I.M., Daetwyler H.D., Goddard M.E. (2020). Effect direction meta-analysis of GWAS identifies extreme, prevalent and shared pleiotropy in a large mammal. Commun. Biol..

[27] Van den Berg I., Xiang R., Jenko J., Pausch H., Boussaha M., Schrooten C., Tribout T., Gjuvsland A.B., Boichard D., Nordbø Ø. (2020). Meta-analysis for milk fat and protein percentage using imputed sequence variant genotypes in 94,321 cattle from eight cattle breeds. Genet. Sel. Evol..

[28] Daetwyler H.D., Capitan A., Pausch H., Stothard P., Van Binsbergen R., Brøndum R.F., Liao X., Djari A., Rodriguez S.C., Grohs C. (2014). Whole-genome sequencing of 234 bulls facilitates mapping of monogenic and complex traits in cattle. Nat. Publ. Gr..

[29] Sanchez M.P., Gion A.G., Croiseau P., Fritz S., Hozé C., Miranda G., Martin P., Leterrier A.B., Letaïef R., Rocha D. (2017). Within-breed and multi-breed GWAS on imputed whole-genome sequence variants reveal candidate mutations affecting milk protein composition in dairy cattle. Genet. Sel. Evol..

[30] Tribout T., Croiseau P., Lefebvre R., Barbat A., Boussaha M., Fritz S., Boichard D., Hoze C., Sanchez M.P. (2020). Confirmed effects of candidate variants for milk production, udder health, and udder morphology in dairy cattle. Genet. Sel. Evol..

[31] Bissonnette N. (2018). Genetic association of variations in the osteopontin gene (SPP1) with lactation persistency in dairy cattle. J. Dairy Sci..

[32] Cole J.B., VanRaden P.M. (2006). Genetic evaluation and best prediction of lactation persistency. J. Dairy Sci..

[33] Walsh S.W., Williams E.J., Evans A.C.O. (2011). A review of the causes of poor fertility in high milk producing dairy cows. Anim. Reprod. Sci..

[34] Nayeri S., Sargolzaei M., Miller S., Schenkel F., Moore S.S., Stothard P. (2017). Genome-wide association study for lactation persistency, female fertility, longevity, and lifetime profit index traits in Holstein dairy cattle. J. Dairy Sci..

[35] Yue S.J., Zhao Y.Q., Gu X.R., Yin B., Jiang Y.L., Wang Z.H., Shi K.R. (2017). A genome-wide association study suggests new candidate genes for milk production traits in Chinese Holstein cattle. Anim. Genet..

[36] Do D.N., Bissonnette N., Lacasse P., Miglior F., Zhao X., Ibeagha-awemu E.M. (2019). Animal Genetics and Genomics: A targeted genotyping approach to enhance the identification of variants for lactation persistency in dairy cows. J. Anim. Sci..

[37] Wang T., Li J., Gao X., Song W., Chen C., Yao D., Ma J. (2020). Genome-wide association study of milk components in Chinese Holstein cows using single nucleotide polymorphism. Livest. Sci..

[38] VanRaden P.M., Van Tassell C.P., Wiggans G.R., Sonstegard T.S., Schnabel R.D., Taylor J.F., Schenke F.S. (2009). Invited review: Reliability of genomic predictions for North American Holstein bulls. J. Dairy Sci..

[39] Sargolzaei M., Chesnais J.P., Schenkel F.S. (2014). A new approach for efficient genotype imputation using information from relatives. BMC Genom..

[40] Larmer S.G., Sargolzaei M., Schenkel F.S. (2014). Extent of linkage disequilibrium, consistency of gametic phase, and imputation accuracy within and across Canadian dairy breeds. J. Dairy Sci..

[41] May K., Sames L., Scheper C., König S. (2021). Genomic loci and genetic parameters for uterine diseases in first-parity Holstein cows and associations with milk production and fertility. J. Dairy Sci..

[42] Klein S.-L., Scheper C., May K., König S. (2020). Genetic and nongenetic profiling of milk β-hydroxybutyrate and acetone and their associations with ketosis in Holstein cows. J. Dairy Sci..

[43] Song Y., Xu L., Chen Y., Zhang L., Gao H., Zhu B., Niu H., Zhang W., Xia J., Gao X. (2016). Genome-wide association study reveals the PLAG1 gene for knuckle, biceps and shank weight in Simmental beef cattle. PLoS ONE.

[44] Purcell S., Neale B., Todd-brown K., Thomas L., Ferreira M.A.R., Bender D., Maller J., Sklar P., De Bakker P.I.W., Daly M.J. (2007). REPORT PLINK: A tool set for whole-genome association and population-based linkage analyses. Am. J. Hum. Genet..

[45] Yang J., Lee S.H., Goddard M.E., Visscher P.M. (2011). REPORT GCTA: A tool for genome-wide complex trait analysis. Am. J. Hum. Genet..

[46] Yang J., Zaitlen N.A., Goddard M.E., Visscher P.M., Price A.L. (2014). Perspective: Advantages and pitfalls in the application of mixed-model association methods. Nat. Publ. Gr..

[47] Prive F., Aschard H., Ziyatdinov A., Blum M.G.B., Timc-imag L. (2018). Genetics and population analysis: Efficient analysis of large-scale genome-wide data with two R packages: Bigstatsr and bigsnpr. Bioinformatics.

[48] Johnson R.C., Nelson G.W., Troyer J.L., Lautenberger J.A., Kessing B.D., Winkler C.A., Brien S.J.O. (2010). Accounting for multiple comparisons in a genome-wide association study (GWAS). BMC Genom..

[49] Li X., Buitenhuis A.J., Lund M.S., Li C., Sun D., Zhang Q., Poulsen N.A., Su G. (2015). Joint genome-wide association study for milk fatty acid traits in Chinese and Danish Holstein populations. J. Dairy Sci..

[50] Goddard M.E., Hayes B.J., Meuwissen T.H.E. (2011). Using the genomic relationship matrix to predict the accuracy of genomic selection. J. Anim. Breed. Genet..

[51] Makanjuola B.O., Miglior F., Abdalla E.A., Schenkel F.S., Baes C.F. (2020). Effect of genomic selection on rate of inbreeding and coancestry and effective population size of Holstein and Jersey cattle populations. J. Dairy Sci..

[52] Wang Z., Shen B., Jiang J., Li J., Ma L. (2016). Effect of sex, age and genetics on crossover interference in cattle. Sci. Rep..

[53] Fonseca P.A.S., Suarez-Vega A., Marras G., Canóvas Á. (2020). GALLO: An R package for genomic annotation and integration of multiple data sources in livestock for positional candidate loci. Giga Sci..

[54] Hu Z.-L., Park C.A., Reecy J.M. (2019). Building a livestock genetic and genomic information knowledgebase through integrative developments of Animal QTLdb and CorrDB. Nucleic Acids Res..

[55] McLaren W., Gil L., Hunt S.E., Riat H.S., Ritchie G.R.S., Thormann A., Flicek P., Cunningham F. (2016). The ensembl variant effect predictor. Genome Biol..

[56] Huang D.W., Sherman B.T., Zheng X., Yang J., Imamichi T., Stephens R., Lempicki R.A. (2009). Extracting biological meaning from large gene lists with DAVID. Curr. Protoc. Bioinform..

[57] Szklarczyk D., Franceschini A., Wyder S., Forslund K., Heller D., Huerta-Cepas J., Simonovic M., Roth A., Santos A., Tsafou K.P. (2015). STRING v10: Protein–protein interaction networks, integrated over the tree of life. Nucleic Acids Res..

[58] Frischknecht M., Bapst B., Seefried F.R., Signer-hasler H., Garrick D., Stricker C., Consortium I., Fries R., Russ I., Sölkner J. (2017). Genome-wide association studies of fertility and calving traits in Brown Swiss cattle using imputed whole-genome sequences. BMC Genom..

[59] Liu L., Zhou J., Chen C.J., Zhang J., Wen W., Tian J., Zhang Z., Gu Y. (2020). GWAS-based identification of new loci for milk yield, fat, and protein in Holstein cattle. Animals.

[60] Ribeiro E.S., Monteiro A.P.A., Bisinotto R.S., Lima F.S., Greco L.F., Ealy A.D. (2016). Conceptus development and transcriptome at preimplantation stages in lactating dairy cows of distinct genetic groups and estrous cyclic statuses. J. Dairy Sci..

[61] Liu A., Wang Y., Sahana G., Zhang Q., Liu L., Lund M.S. (2017). Genome-wide association studies for female fertility traits in Chinese and Nordic Holsteins. Sci. Rep..

[62] Jiang J., Ma L., Prakapenka D., Vanraden P.M., Cole J.B., Cole J.B. (2019). A large-scale genome-wide association study in US Holstein Cattle. Front. Genet..

[63] Albarran-Portillo B., Pollott G.E. (2013). The relationship between fertility and lactation characteristics in Holstein cows on United Kingdom commercial dairy farms. J. Dairy Sci..

[64] Muir B.L., Fatehi J., Schaeffer L.R. (2004). Genetic relationships between persistency and reproductive performance in first-lactation Canadian Holsteins. J. Dairy Sci..

[65] Jakobsen J.H., Madsen P., Jensen J., Pedersen J., Christensen L.G., Sorensen D.A. (2002). Genetic parameters for milk production and persistency for Danish Holsteins estimated in random regression models using REML. J. Dairy Sci..

[66] Yamazaki T., Hagiya K., Takeda H., Yamaguchi S., Osawa T., Nagamine Y. (2014). Genetic correlations among female fertility, 305-day milk yield and persistency during the first three lactations of Japanese Holstein cows. Livest. Sci..

[67] Santos D.J.A., Cole J.B., Null D.J., Byrem T.M., Ma L. (2018). Genetic and nongenetic profiling of milk pregnancy-associated glycoproteins in Holstein cattle. J. Dairy Sci..

[68] Le Guillou S., Sdassi N., Laubier J., Passet B., Vilotte M., Castille J., Polyte J., Jaffre F., Cribiu E., Vilotte J. (2012). Overexpression of miR-30b in the developing mouse mammary gland causes a lactation defect and delays involution. PLoS ONE.

[69] Law R.H.P., Zhang Q., Mcgowan S., Buckle A.M., Silverman G.A., Wong W., Rosado C.J., Chris G., Pike R.N., Bird P.I. (2006). An overview of the serpin superfamily. Genome Biol..

[70] De Camargo G.M.F., Aspilcueta-borquis R.R., Cardoso D.F., Santos D.J.A. (2015). Prospecting major genes in dairy buffaloes. BMC Genom..

[71] Soares R.A.N., Vargas G., Duffield T., Schenkel F., Squires J. (2021). Genome-wide association study and functional analyses for clinical and subclinical ketosis in Holstein cattle. J. Dairy Sci..

[72] Oliveira H.R., Lourenco D.A.L., Masuda Y., Misztal I., Tsuruta S., Jamrozik J., Brito L.F., Silva F.F., Cant J.P., Schenkel F.S. (2019). Single-step genome-wide association for longitudinal traits of Canadian Ayrshire, Holstein, and Jersey dairy cattle. J. Dairy Sci..

[73] Clancey E., Kiser J.N., Moraes J.G.N., Dalton J.C., Spencer T.E., Neibergs H.L. (2019). Genome-wide association analysis and gene set enrichment analysis with SNP data identify genes associated with 305-day milk yield in Holstein dairy cows. Anim. Genet..

[74] Atashi H., Crowe M., Salavati M., De Koster J., Ehrlich J., Crowe M., Opsomer G., Hostens M. (2020). Genome-wide association for milk production and lactation curve parameters in Holstein dairy cows. J. Anim. Breed. Genet..

[75] Raven L., Cocks B.G., Hayes B.J. (2014). Multibreed genome wide association can improve precision of mapping causative variants underlying milk production in dairy cattle. BMC Genom..

[76] Pradeep J., Monika S., Ankita S., Ks U., Amit K., Ashok M., Bp M., Sandeep M., Rs K., Kaushik J. (2014). Expression analysis of solute carrier (SLC2A) genes in milk derived mammary epithelial cells during different stages of lactation in Sahiwal (*Bos indicus*) cows advances in dairy research. Adv. Dairy Res..

[77] Banos G., Clark E.L., Id S.J.B., Dutta P., Id G.B., Arsenos G., Hume D.A., Id A.P. (2019). Genetic and genomic analyses underpin the feasibility of concomitant genetic improvement of milk yield and mastitis resistance in dairy sheep. PLoS ONE.

[78] Meredith B.K., Kearney F.J., Finlay E.K., Bradley D.G., Fahey A.G., Berry D.P., Lynn D.J. (2012). Genome-wide associations for milk production and somatic cell score in Holstein-Friesian cattle in Ireland. BMC Genet..

[79] Buitenhuis B., Janss L.L.G., Poulsen N.A., Larsen L.B., Larsen M.K., Sørensen P. (2014). Genome-wide association and biological pathway analysis for milk-fat composition in Danish Holstein and Danish Jersey cattle. BMC Genom..

[80] Buitenhuis B., Poulsen N.A., Gebreyesus G., Larsen L.B. (2016). Estimation of genetic parameters and detection of chromosomal regions affecting the major milk proteins and their post translational modifications in Danish Holstein and Danish Jersey cattle. BMC Genet..

[81] Cole J.B., Wiggans G.R., Ma L., Sonstegard T.S., Lawlor T.J., Crooker B.A., Van Tassell C.P., Yang J., Wang S., Matukumalli L.K. (2011). Genome-wide association analysis of thirty one production, health, reproduction and body conformation traits in contemporary US Holstein cows. BMC Genom..

[82] Zhou J., Liu L., Chen C.J., Zhang M., Lu X., Zhang Z. (2019). Genome-wide association study of milk and reproductive traits in dual-purpose Xinjiang Brown cattle. BMC Genom..

[83] Zhang X., Li C., Li X., Liu Z., Ni W., Cao Y., Yao Y., Islamov E. (2019). Association analysis of polymorphism in the NR6A1 gene with the lumbar vertebrae number traits in sheep. Genes Genom..

[84] Klomtong P., Chaweewan K., Phasuk Y., Duangjinda M. (2015). genetic differentiation in Thai native, wild boars, and Duroc and Chinese Meishan pigs. Genet. Mol. Res..

[85] Tokunaga M., Inoue M., Jiang Y., Barnes R.H., Buchner D.A., Chun T.-H. (2014). Fat depot-specific gene signature and ECM remodeling of Sca1high adipose-derived stem cells. Matrix Biol..

[86] Buitenhuis B., Poulsen N.A., Larsen L.B., Sehested J. (2015). Estimation of genetic parameters and detection of quantitative trait loci for minerals in Danish Holstein and Danish Jersey milk. BMC Genet..

[87] Grisart B., Coppieters W., Farnir F., Karim L., Ford C., Berzi P., Cambisano N., Mni M., Reid S., Simon P. (2002). Positional candidate cloning of a QTL in dairy cattle: Identification of a missense mutation in the bovine DGAT1 gene with major effect on milk yield and composition. Genome Res..

[88] Cai Z., Guldbrandtsen B., Lund M.S., Sahana G. (2018). Dissecting closely linked association signalsin combination with the mammalianphenotype database can identify candidategenes in dairy cattle. BMC Genet..

[89] Palombo V., Milanesi M., Sgorlon S., Capomaccio S., Mele M., Nicolazzi E. (2018). Genome-wide association study of milk fatty acid composition in Italian Simmental and Italian Holstein cows using single nucleotide polymorphism arrays. J. Dairy Sci..

[90] Frischknecht M., Pausch H., Bapst B., Signer-hasler H., Flury C., Garrick D., Stricker C., Fries R., Gredler-grandl B. (2017). Highly accurate sequence imputation enables precise QTL mapping in Brown Swiss cattle. BMC Genom..

[91] Ning C., Kang H., Zhou L., Wang D., Wang H., Wang A., Fu J. (2017). Performance gains in genome-wide association studies for longitudinal traits via modeling time-varied effects. Sci. Rep..

[92] Fang Z.-H., Pausch H. (2019). Multi-trait meta-analyses reveal 25 quantitative trait loci for economically important traits in Brown Swiss cattle. BMC Genom..

[93] Cai Z., Dusza M., Guldbrandtsen B., Lund M.S., Sahana G. (2020). Distinguishing pleiotropy from linked QTL between milk production traits and mastitis resistance in Nordic Holstein cattle. Genet. Sel. Evol..

[94] Fonseca I., Cardoso F.F., Higa R.H., Giachetto P.F., Brandão H.M., Brito M.A.V.P., Ferreira M.B.D., Guimarães S.E.F., Martins M.F. (2015). Gene expression profile in zebu dairy cows (*Bos taurus indicus*) with mastitis caused by *Streptococcus agalactiae*. Livest. Sci..

[95] Koh Y., Peiris H., Vaswani K., Almughlliq F., Meier S., Burke C., Mitchell M. (2020). Exosomes from dairy cows of divergent fertility; Action on endometrial cells. J. Reprod. Immunol..

[96] Marcos-Carcavilla A., Calvo J.H., González C., Moazami-Goudarzi K., Laurent P., Bertaud M., Hayes H., Alves M.E.F., Serrano M. (2007). Short communication: IL-1 family members as possible candidate genes affencting economically important traits in cattle. Span. J. Agric. Res..

[97] Yu G.I., Song D.K., Shin D. (2020). Associations of IL1RAP and IL1RL1 gene polymorphisms with obesity and inflammation mediators. Inflamm. Res..

[98] Ogorevc J., Kunej T., Razpet A., Dovc P. (2009). Database of cattle candidate genes and genetic markers for milk production and mastitis. Anim. Genet..

[99] Kaniyamattam K., De Vries A. (2014). Agreement between milk fat, protein, and lactose observations collected from the Dairy Herd Improvement Association (DHIA) and a real-time milk analyzer. J. Dairy Sci..

[100] Mastrangelo S., Ben S., Elena J., Gianluca C., Moscarelli A., Boussaha M., Montedoro M., Pilla F., Cassandro M. (2020). Genome-wide detection of signatures of selection in three Valdostana cattle populations. J. Anim. Breed. Genet..

[101] Liu J.J., Liang A.X., Campanile G., Plastow G., Zhang C., Wang Z., Salzano A., Gasparrini B. (2018). Genome-wide association studies to identify quantitative trait loci affecting milk production traits in water buffalo. J. Dairy Sci..

[102] Raschia M.A., Nani J.P., Carignano H.A., Amadio A.F., Maizon D.O., Poli M.A., Nacional I., Agropecuaria D.T., De Genética I., Favret E.A. (2020). Weighted single-step genome-wide association analyses for milk traits in Holstein and Holstein x Jersey crossbred dairy cattle. Livest. Sci..

[103] Jena M.K., Mohanty A.K. (2017). New insights of mammary gland during different stages of development. Asian J. Pharm. Clin. Res..

[104] Lin S., Wan Z., Zhang J., Xu L., Han B., Sun D. (2020). Genome-wide association studies for the concentration of albumin in colostrum and serum in Chinese Holstein. Animals.

[105] Jiménez-González V., Ogalla-García E., García-Quintanilla M., García-Quintanilla A. (2019). Deciphering GRINA/Lifeguard1: Nuclear location, ca2+ homeostasis and vesicle transport. Int. J. Mol. Sci..

[106] Kaufmann M., Feijis K., Lüscher B. (2014). Endogenous ADP-Ribosylation. https://link.springer.com/chapter/10.1007/82_2014_379.

[107] Zhou C., Li C., Cai W., Liu S., Yin H., Shi S., Zhang Q. (2019). Genome-wide association study for milk protein composition traits in a Chinese Holstein population using a single-step approach. Front. Genet..

[108] Do D.N., Bissonnette N., Lacasse P., Miglior F., Sargolzaei M., Zhao X. (2017). Genome-wide association analysis and pathways enrichment for lactation persistency in Canadian Holstein cattle. J. Dairy Sci..

[109] Mohan T., Deng L., Wang B.-Z. (2017). CCL28 chemokine: An anchoring point bridging innate and adaptive immunity. Int. Immunopharmacol..

[110] Tomazi T., Gonçalves J.L., Barreiro J.R., Arcari M.A., Dos Santos M.V. (2015). Bovine subclinical intramammary infection caused by coagulase-negative staphylococci increases somatic cell count but has no effect on milk yield or composition. J. Dairy Sci..

[111] Huang W., Peñagaricano F., Ahmad K.R., Lucey J.A., Weigel K.A., Khatib H. (2012). Association between milk protein gene variants and protein composition traits in dairy cattle. J. Dairy Sci..

[112] Yang F., Zhang M., Rong Y., Liu Z., Yang S., Zhang W. (2020). A novel SNPs in alpha-lactalbumin gene effects on lactation traits in Chinese Holstein dairy cows. Animals.

[113] Raven L., Cocks B.G., Kemper K.E., Chamberlain A.J., Vander C.J., Michael J., Hayes B.J. (2016). Targeted imputation of sequence variants and gene expression profiling identifies twelve candidate genes associated with lactation volume, composition and calving interval in dairy cattle. Mamm. Genome.

[114] Du C., Deng T., Zhou Y., Ye T., Zhou Z., Zhang S., Shao B., Wei P., Sun H., Khan F.A. (2019). Systematic analyses for candidate genes of milk production traits in water buffalo (*Bubalus Bubalis*). Anim. Genet..

[115] Laodim T., Elzo M.A., Koonawootrittriron S., Suwanasopee T., Jattawa D. (2019). Genomic-polygenic and polygenic predictions for milk yield, fat yield, and age at first calving in Thai multibreed dairy population using genic and functional sets of genotypes. Livest. Sci..

[116] Mrode R., Ojango J.M.K., Okeyo A.M., Mwacharo J.M. (2019). Genomic selection and use of molecular tools in breeding programs for indigenous and crossbred cattle in developing countries: Current status and future prospects. Front. Genet..

[117] Rong L., Qing-Zhang L., Jian-Guo H., Li-min L., Xue-Jun G. (2013). Proteomic identification of differentially expressed proteins in *Vaccaria segetalis*-treated dairy cow mammary epithelial cells. J. Northeast Agric. Univ..

