# Supplementary material for: Genomewide Association Analyses of Lactation Persistency and Milk Production Traits in Holstein Cattle Based on Imputed Whole-Genome Sequence Data
Source: Genes (Basel). 2021 Nov 19;12(11):1830. doi: 10.3390/genes12111830 (PMC8624223; doi:10.3390/genes12111830)
Supplement: Supplementary file 1 [file genes-12-01830-s001.zip › supplementary_files/Figure S1.pdf]

**(a) MILK**

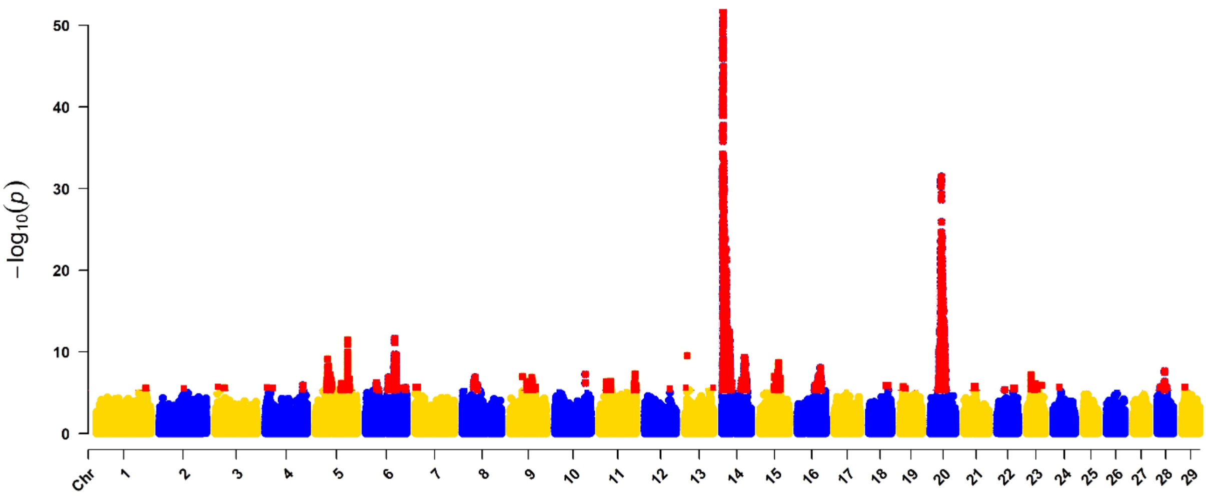


**(b) FAT**

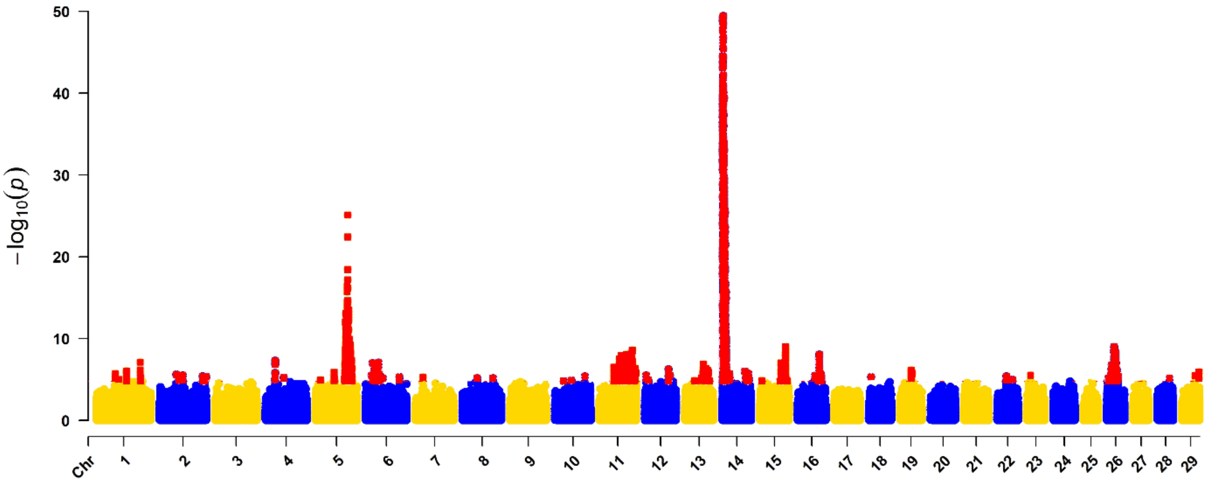


**(c) FAT%**

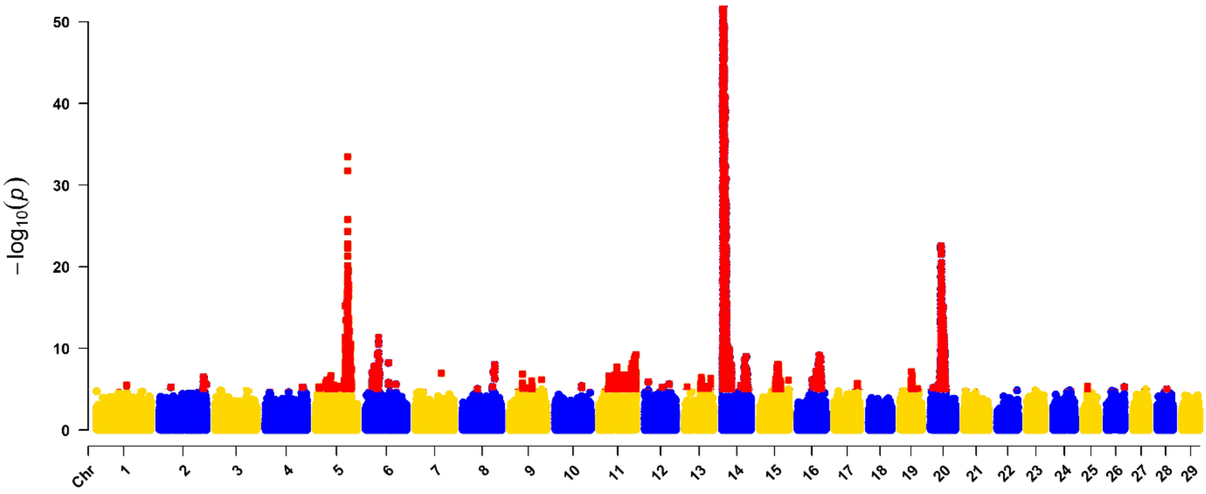

**(d) PROT**

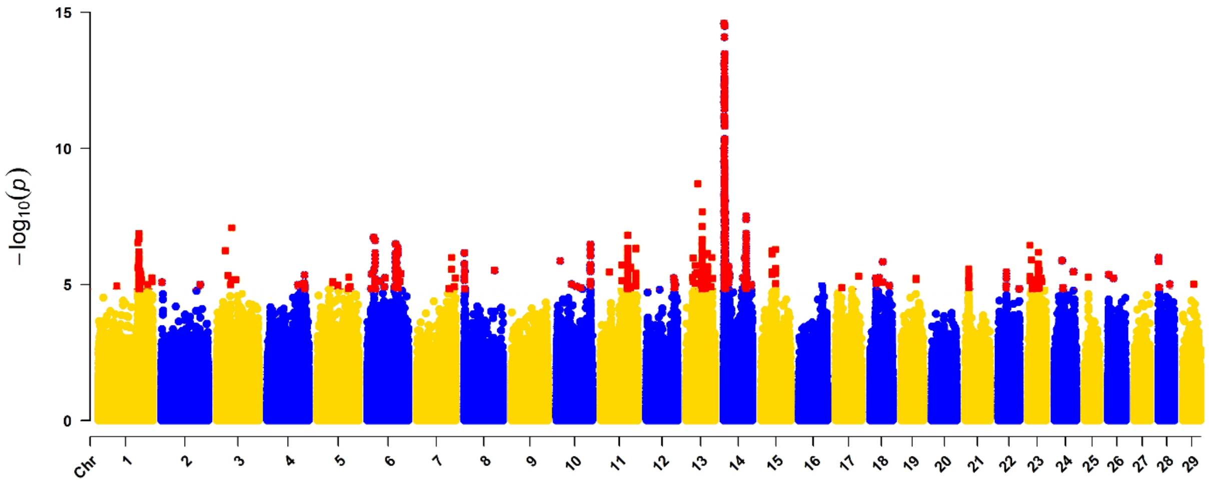


**(e) PROT%**

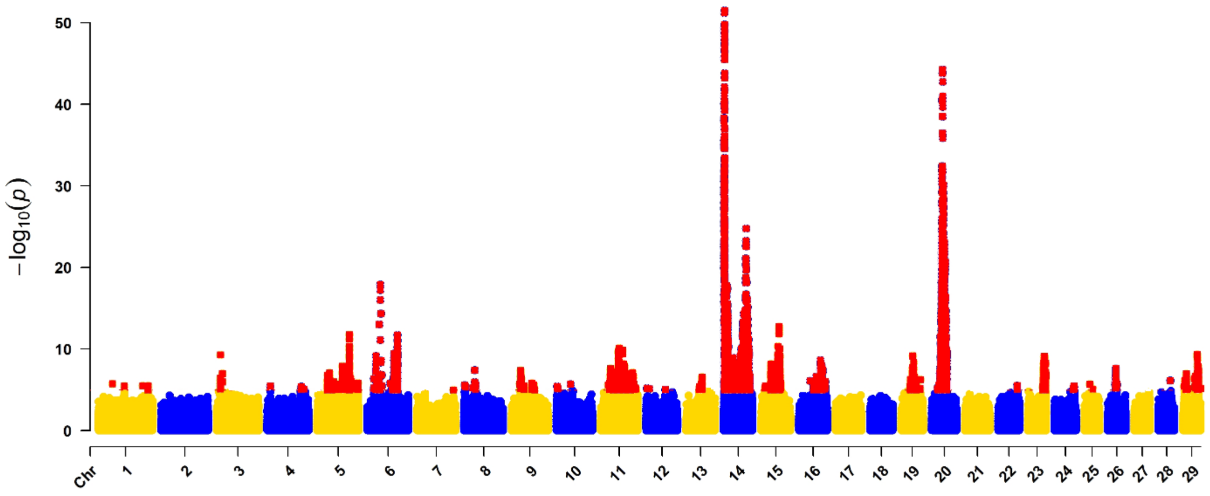


**Supplementary File S1.** Manhattan plots with truncated Y-axis for the GWAS results for milk yield (MILK), fat yield (FAT), fat percentage (FAT%), protein yield (PROT) and protein percentage (PROT%) based on imputed whole-genome sequence data. Statistically significant SNP are represented by red dots.
