# Supplementary figures and images for: Genomewide Association Analyses of Lactation Persistency and Milk Production Traits in Holstein Cattle Based on Imputed Whole-Genome Sequence Data
Source: Genes (Basel). 2021 Nov 19;12(11):1830. doi: 10.3390/genes12111830 (PMC8624223; doi:10.3390/genes12111830)

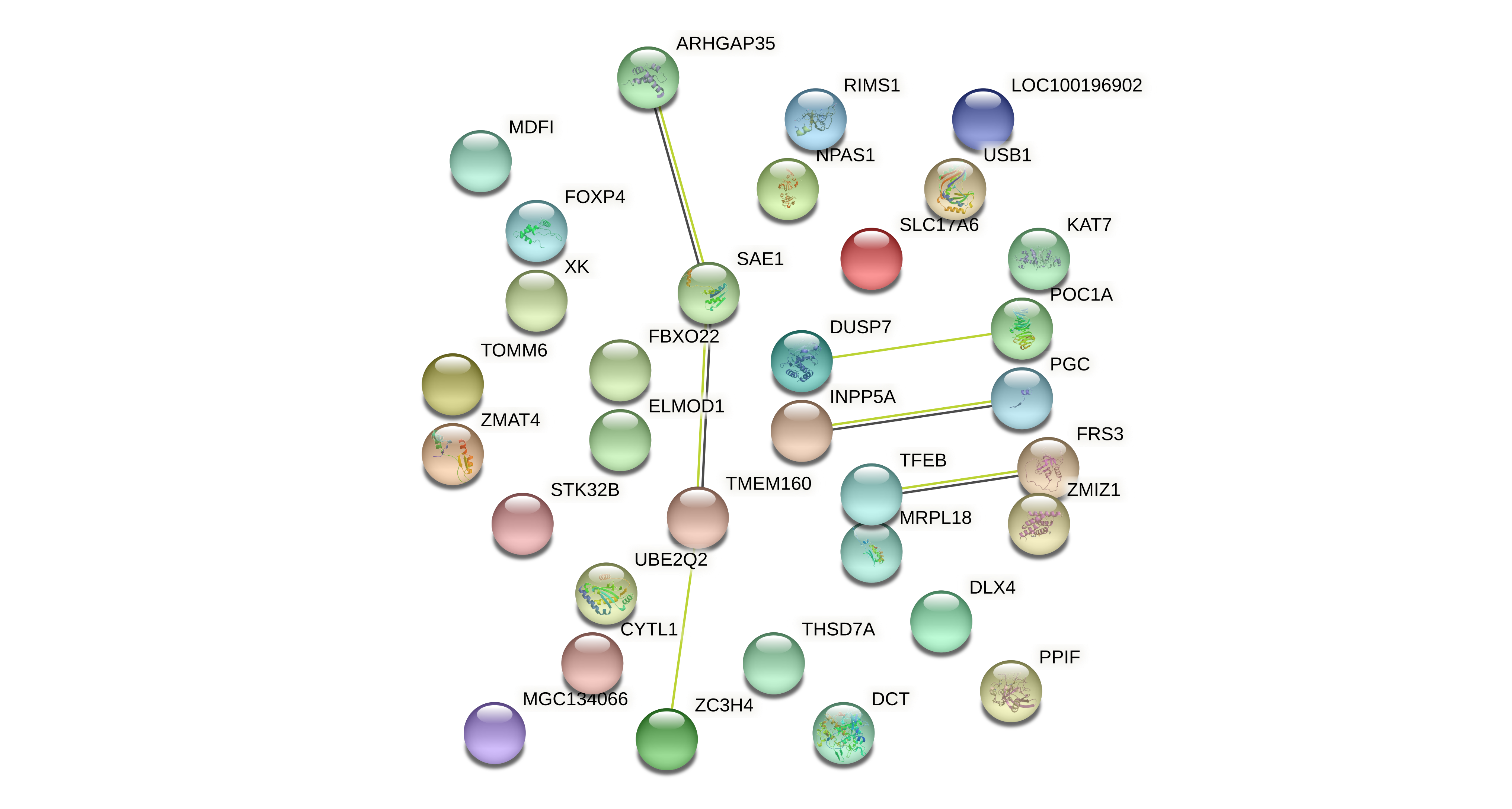

Supplement: Supplementary file 1 [file genes-12-01830-s001.zip › supplementary_files/Figure S2.png]
